# The physiological and molecular responses of potato tuberization to projected future elevated temperatures

**DOI:** 10.1093/plphys/kiae664

**Published:** 2024-12-17

**Authors:** Abigail M Guillemette, Guillian Hernández Casanova, John P Hamilton, Eva Pokorná, Petre I Dobrev, Václav Motyka, Aaron M Rashotte, Courtney P Leisner

**Affiliations:** Department of Biological Sciences, Auburn University, Auburn, AL 36849, USA; School of Plant and Environmental Sciences, Virginia Polytechnic Institute and State University, Blacksburg, VA 24061, USA; Department of Crop and Soil Sciences, Center for Applied Genetic Technologies, University of Georgia, Athens, GA 30602, USA; Department of Forest Tree Species Biology and Breeding, Forestry and Game Management Research Institute, Jíloviště 25202, Czech Republic; Laboratory of Hormonal Regulations in Plants, Institute of Experimental Botany of the Czech Academy of Sciences, Prague 6, Lysolaje 16502, Czech Republic; Laboratory of Hormonal Regulations in Plants, Institute of Experimental Botany of the Czech Academy of Sciences, Prague 6, Lysolaje 16502, Czech Republic; Laboratory of Hormonal Regulations in Plants, Institute of Experimental Botany of the Czech Academy of Sciences, Prague 6, Lysolaje 16502, Czech Republic; Department of Biological Sciences, Auburn University, Auburn, AL 36849, USA; Department of Biological Sciences, Auburn University, Auburn, AL 36849, USA; School of Plant and Environmental Sciences, Virginia Polytechnic Institute and State University, Blacksburg, VA 24061, USA

## Abstract

Potato (*Solanum tuberosum* L.) is one of the most important food crops globally and is especially vulnerable to heat stress. However, substantial knowledge gaps remain in our understanding of the developmental mechanisms associated with tuber responses to heat stress. This study used whole-plant physiology, transcriptomics, and phytohormone profiling to elucidate how heat stress affects potato tuber development. When plants were grown in projected future elevated temperature conditions, abscisic acid (ABA) levels decreased in leaf and tuber tissues, whereas rates of leaf carbon assimilation and stomatal conductance were not significantly affected compared to those plants grown in historical temperature conditions. While plants grown in projected future elevated temperature conditions initiated more tubers per plant on average, there was a 66% decrease in mature tubers at the final harvest compared to those plants grown in historical temperature conditions. We hypothesize that reduced tuber yields at elevated temperatures are not due to reduced tuber initiation, but due to impaired tuber filling. Transcriptomic analysis detected significant changes in the expression of genes related to ABA response, heat stress, and starch biosynthesis. The tuberization repressor genes SELF-PRUNING 5G (*StSP5G*) and CONSTANS-LIKE1 (*StCOL1*) were differentially expressed in tubers grown in elevated temperatures. Two additional known tuberization genes, IDENTITY OF TUBER 1 (*StIT1*) and TIMING OF CAB EXPRESSION 1 (*StTOC1*), displayed distinct expression patterns under elevated temperatures compared to historical temperature conditions but were not differentially expressed. This work highlights potential gene targets and key developmental stages associated with tuberization to develop potatoes with greater heat tolerance.

## Teaser

Chronic heat stress reduces potato yield by inhibiting tuber filling, possibly due to decreased abscisic acid levels and increased gene expression of known repressors of tuberization.

## Introduction

Since the industrial revolution, average global temperatures have risen by ∼1.1 °C, and unless carbon dioxide (CO_2_) emissions are substantially diminished, global surface temperatures are expected to continue to increase by an additional 1.5 to 8 °C by the next century (IPCC 2021). These higher-than-optimum temperatures have already been shown to negatively affect productivity and yield in many major crop plants, including potato (*Solanum tuberosum* L.) (Dahal et al. 2019). Potatoes are the fourth most important food crop globally with a total production of 374 million metric tons in 2022 (FAO 2022). It is estimated that climate change, including temperature fluctuations and heat stress, has the potential to reduce potato production by up to 18% to 32% by mid-century (Hijmans 2003). As a staple crop in many countries, understanding how elevated temperatures (ElevTs) associated with climate change affect potato development and yield is of utmost importance for future global food security (de Haan and Rodriguez 2016).

Diploid progenitors of modern tetraploid potatoes are native to the more temperate climate of the Andes Mountains of South America (Ortiz 2001), with optimal temperatures for tuber growth being 14 to 22 °C (Van Dam et al. 1996). Potato plants grown under temperatures even moderately higher than this range (≥25 °C) demonstrate a shift in biomass allocation toward the aboveground plant, leading to longer stems, more leaves, and decreased tuber yield (Hancock et al. 2014). Although the degree of this response varies among potato cultivars, even relatively heat-tolerant cultivars can experience significant yield loss. Additionally, yield loss is exacerbated the earlier in development the heat stress occurs (Rykaczewska 2013, 2015). There is a significant knowledge gap, however, surrounding the mechanism by which ElevTs affect tuberization signaling, tuber development, and, in turn, potato yield.

Potato tubers develop from a stolon, or underground stem, originating from axillary buds at the base of the main stem. The development of a stolon into a tuber is caused by changes in the carbohydrate metabolism of the plant in response to environmental cues such as photoperiod and temperature (Kondhare et al. 2021). These cues cause a cascade of internal signals controlling tuberization, and the process is characterized by 3 separate stages: tuber initiation, tuber filling, and maturation (Obidiegwu et al. 2015). During tuber initiation, the stolon apical tip ceases elongation and begins radial cell expansion and division, driven by transport and deposition of sucrose synthesized in the leaves during photosynthesis (Zierer et al. 2021). During the subsequent filling and maturation stage, the deposition of sucrose and other metabolites in the stolon continues until the tuber reaches maturity.

Tuberization is mediated by complex interactions between molecular tuberization signals and environmental cues (Dutt et al. 2017). The most well-studied gene responsible for regulating tuberization is *S. tuberosum SELF-PRUNING 6A (StSP6A*), a *FLOWERING LOCUS T* (*FT*) homolog and mobile protein that induces sucrose transport from the leaves to tubers (Navarro et al. 2011). During tuber initiation, StSP6A forms the tuberigen activation complex in the stolon tips with *FLOWERING LOCUS D* (*FD*)-like proteins *StFDL1a* and *StFDL1b*, which are basic leucine zipper transcription factors (Teo et al. 2017). *StSP6A* is part of the phosphatidylethanolamine-binding protein (PEBP) gene family along with 14 other genes related to flowering/tuberization (Zhang et al. 2022). Under heat stress, it is known that during early stages of tuberization, *StSP6A* is inhibited posttranscriptionally through RNA-based interference by a small RNA called SUPPRESSING EXPRESSION OF SP6A, while transcriptional regulation of *StSP6A* is the main regulation process in later stages of tuber development under heat stress (Lehretz et al. 2019; Park et al. 2022). Studies using *StSP6A* overexpression lines found increased tuber count under high temperatures compared to wild-type (WT) plants, but yield/tuber weight was still significantly decreased compared to WT in ambient temperature (AmbT) (Park et al. 2022). This indicates that while *StSP6A* plays a significant role in tuber formation, other genes are responsible for the continued growth and development of tubers to maturity when grown in ElevTs.

The CONSTANS LIKE (*StCOL1*) and SELF-PRUNING 5G (*StSP5G*) inhibitory pathway is known to be an important suppressor of tuberization. Accumulation of the StCOL1 protein activates *StSP5G*, an inhibitor of *StSP6A* (Valverde 2011; Abelenda et al. 2016). Both genes are known to be expressed primarily in leaves, although their expression has also been found in tubers (Park et al. 2022). Another inhibitor of *StSP6A* is TIMING OF CAB EXPRESSION 1 (TOC1), a circadian clock gene known to interact with the promoter of *StSP6A*. Studies have shown that *TOC1* has increased expression in ElevT and interacts directly with the StSP6A tuberization signal, suppressing its positive feed-forward regulation in stolons (Morris et al. 2019).

Despite recent developments in understanding the effects of heat stress on potato yield, the exact molecular mechanisms controlling tuberization under ElevTs remain unknown. Furthermore, few studies have observed developing tubers under chronic heat stress that mimic realistic projected future climate conditions. To address this knowledge gap, we employed dynamically downscaled global climate projections to determine anticipated future growth conditions for a major potato production region for the mid-21st century to study the effects of ElevT on potato development. In this controlled growth chamber experiment, we investigated the physiological and molecular changes of both source and sink tissue over time under ambient and ElevT conditions. Findings from this study have the potential to advance our understanding of the physiological mechanisms associated with potato tuberization in ElevT and identify key tuberization genes that may help enhance potato resilience to future ElevTs.

## Results

### ElevT impacts on leaf physiology

**Figure 1.**
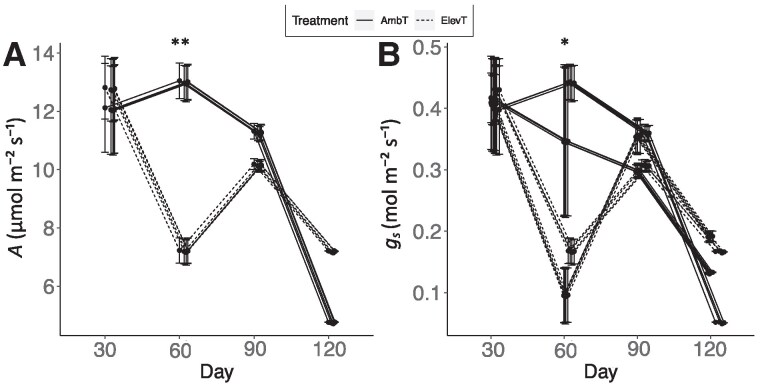
Gas exchange measurements of potato plants grown in AmbT and ElevT conditions. Rates of **A)** carbon assimilation (*A*, measured in umol m^−2^ s^−1^) and **B)** stomatal conductance (*g_s_*, measured in mol m^−2^ s^−1^) of potato plants are shown. Solid lines represent AmbT plants while dashed lines represent ElevT. Error bars represent standard error between biological replicates (*n* = 2). Asterisks indicate significant differences between AmbT and ElevT from pairwise *t*-tests at each time point (*P < 0.05; **P < 0.01).

To understand the effects of ElevT on potato leaf physiology, gas exchange of the youngest mature leaves was measured to determine rates of carbon assimilation (*A*) and stomatal conductance (*g_s_*) at each time point. Rates of *A* and *g_s_* were not significantly different between treatment groups except at 60 d where the plants grown in projected ElevT conditions had significantly lower rates of both *A* and *g_s_* than plants grown in the historical temperature conditions (which will be referred to as AmbT) (Fig. 1). Although plants from both AmbT and ElevT treatment groups were initially watered equally, at the 60-d sampling date, ElevT plants were noticed to have visibly drier soil than the AmbT plants, after which all plants were watered according to the soil moisture level. It is possible that changes in *A* and *g_s_* seen at this sampling point are due to this drying effect and an increased rate of evapotranspiration under higher temperatures (Singh et al. 2015). Both *A* and *g_s_* rates in the ElevT plants returned to AmbT levels at 90 and 120 d when both treatment groups were sufficiently watered, likely attributing the negative effects seen at 60 d to water deficit.

Chlorophyll fluorescence was also measured at each time point with the maximum PSII quantum efficiency (*F_v_*/*F_m_*) used as a proxy for rates of dark-induced leaf senescence (Zwack et al. 2016). There were no significant differences in senescence rates between treatment groups except at 60 d, where the ElevT plants had 44.7% higher *F_v_*/*F_m_* measurements than AmbT leaves after 72 h (Supplementary Fig. S1). This indicates a possible slower rate of senescence in the ElevT plants at 60 d, which is the same time point when gas exchange measurements showed a significant decline in ElevT plants, likely due to the mild drought stress they were receiving. Additionally, there were no significant differences in total leaf chlorophyll content between plants grown in AmbT and ElevT at any time points measured (Supplementary Fig. S2).

### Biomass and yield

**Figure 2.**
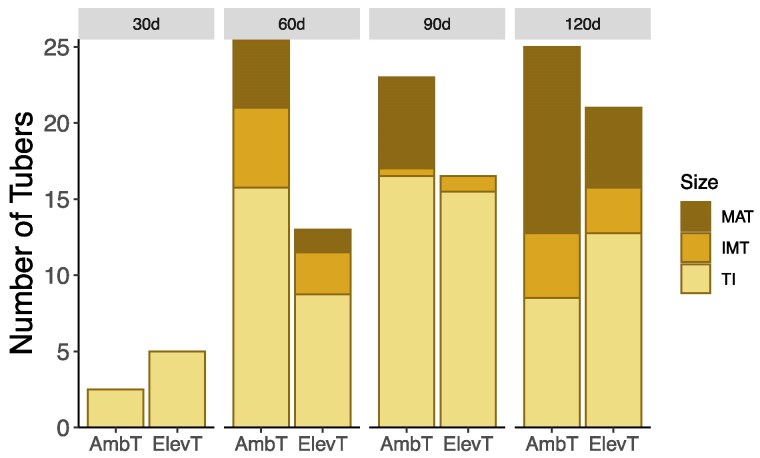
Stacked bar plot showing the average number of tubers per plant per treatment for each size class. Counts are shown as average number of tubers per treatment per size class. Two plants were harvested per chamber at 30 and 90 d, and 4 plants per chamber were harvested at 60 and 120 d, respectively.

At each collection time point, tubers were harvested from all chambers and assigned a size class based on weight: tuber initials (TIs), immature tubers (IMTs), and mature tubers (MATs) (Supplementary Fig. S3). TIs were collected at each time point in both AmbT and ElevT conditions, while IMT and MAT were not found at the 30-d collection point in either treatment (Fig. 2). There were no significant differences in the total number of TI or IMT collected between AmbT and ElevT treatments across developmental time points (Supplementary Table S1). At 90 d, no MATs from ElevT plants were collected, and at 120 d, there were significantly fewer average number of MAT per plant in ElevT (2.63 ± 1.8) compared to AmbT (6.12 ± 1.1) (Supplementary Table S1).

**Figure 3.**
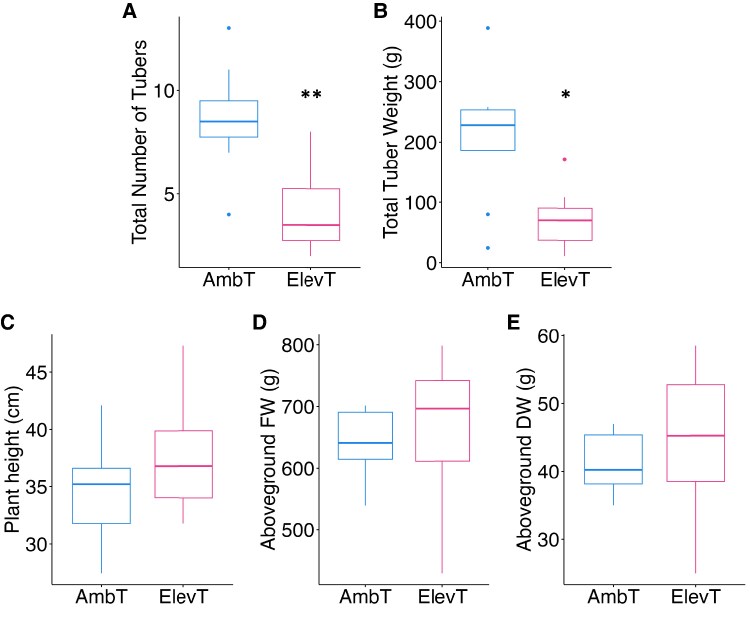
Final harvest data from potato plants grown in AmbT and ElevT conditions. Box plots showing the **A)** average total number of tubers per plant, **B)** average total tuber weight per plant, **C)** average plant height (cm), **D)** average aboveground FW per plant (g), and **E)** average aboveground DW per plant (g) for each treatment group at 120 d. TIs were excluded from the final yield measurements. Values represent the average of 4 plants per chamber (*n* = 2). Centerline, median; box limits, upper and lower quartiles; whiskers, 1.5× interquartile range; points, potential outliers. Asterisks indicate significant differences between AmbT and ElevT from pairwise *t*-tests (*P < 0.05; **P < 0.01).

At the final harvest (120 d), the average number of tubers per plant and average tuber weight per plant were determined for all tubers above 0.6 g (combining both IMT and MAT). Plants grown in ElevT conditions had significantly decreased tuber yield (both count and weight) compared to AmbT plants (Fig. 3, A and B). For tuber count, an average of 12.25 MATs per plant was collected from AmbT plants, while in ElevT, a significant decrease to 5.24 MAT per plant (57% decrease; Fig. 3A) was observed. Regarding tuber weight, AmbT plants had a mean tuber weight per plant of 210 ± 73 g compared to 72.3 ± 13.5 g for ElevT plants (66% decrease in ElevT; Fig. 3B). Plant height, aboveground fresh weight (FW), and aboveground dry weight (DW) were not significantly different between treatment groups, although the aboveground biomass of ElevT plants was slightly more than AmbT plants by up to 8% in both fresh and DW (Fig. 3, C to E).

### Phytohormone content

**Figure 4.**
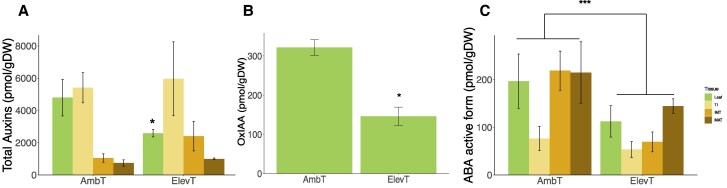
Total levels of phytohormones in potato tissues in AmbT and ElevT conditions. **A)** Total auxin levels (pmol/g DW) across tissues; **B)** levels of OxIAA (pmol/g DW) in leaves; and **C)** levels of the ABA active form across tissues. Values represent levels pooled from 90- and 120-d-old plants exhibiting similar trends. Asterisks indicate significant differences between AmbT and ElevT for the corresponding tissues from pairwise *t*-tests (*P < 0.05; ***P < 0.0001). Error bars are standard errors between biological replicates (*n* = 2).

To investigate changes in signaling in plants grown under ElevT, we analyzed endogenous phytohormone content of all tissues (leaf, TI, IMT, and MAT) using LC/MS averaged across the 90- and 120-d time points. In total, 49 phytohormone metabolite forms could be detected: 10 auxins, 23 cytokinins, 5 abscisic acids (ABAs), 6 jasmonic acids (JAs), and 5 other phenolic compounds including salicylic acid (SA) (Supplementary Table S2, A to D), with gibberellic acid (GA) forms below detectable levels. There were no significant differences in total levels of cytokinins, JAs, or SA between treatment groups, likely due to large variation among biological replicates (Supplementary Table S2, A to D). Total auxin levels were significantly decreased in leaves, with a 46% decrease in leaves grown in ElevT conditions compared to AmbT conditions (Fig. 4A). When specific auxin metabolites were compared, the total difference was likely attributable to significantly lower levels of oxo-indole-3-acetic acid (OxIAA) in leaves (55% decreased from AmbT to ElevT) (Fig. 4B).

Due to its known role as both a stress hormone and its involvement in promoting tuber induction, the levels of ABA and its metabolites (ABAs) were also analyzed. The levels of ABA were significantly lower in ElevT compared to AmbT when averaged across all tissue types, with levels ranging between 76 to 219 pmol/g DW in AmbT and 53 to 144 pmol/g DW in ElevT (Fig. 4C). The decrease in ABA levels is especially evident in IMT in which ElevT samples had a 68% decrease of ABA compared to AmbT (Fig. 4C).

### Differential gene expression analysis

**Figure 5.**
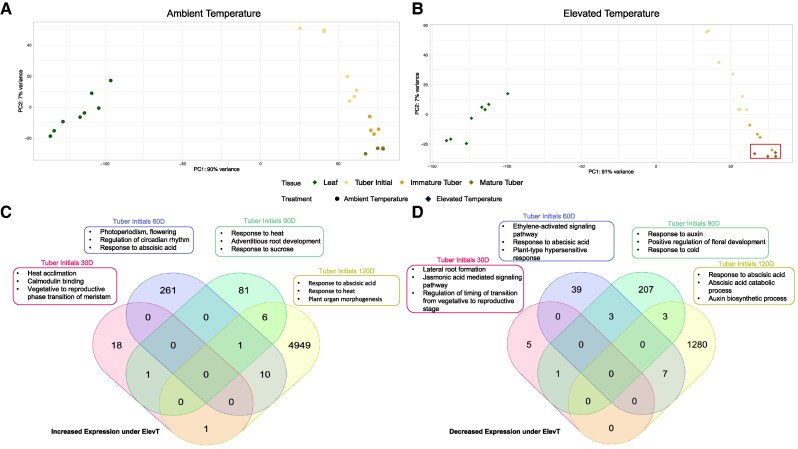
PCA and Venn diagram of expression patterns across RNA-seq libraries. PCA with libraries colored by tissue type for AmbT **A)** and ElevT **B)**. Graphs were produced using *prcomp* in R. **C)** Venn diagram highlighting the number of shared increased DEGs between developmental stages of tubers under both AmbT and ElevT, with selected GO terms of DEGs at each time point. **D)** Venn diagram highlighting the number of shared decreased DEGs between developmental stages of tubers under both AmbT and ElevT, with selected GO terms of DEGs at each time point. Red box indicates area of the plot where the IMT and MAT samples are grouped together.

To examine differential expression of genes across the potato transcriptome, RNA-sequencing (RNA-seq) was performed on total RNA extracted from all tissue samples. Libraries were aligned to the tetraploid Atlantic Cultivar (ATL_v3) *S. tuberosum* genome, and mapping alignment statistics were assessed (Supplementary Table S3). Principal component analysis (PCA) showed that most of the variation in RNA-seq libraries was accounted for by organ type, with differences between leaves and tubers explaining 91% of the variance (Supplementary Fig. S4). To identify the effects of each treatment on gene expression, a separate PCA was conducted for the samples within each treatment group. From the PCA of AmbT samples, distinct clusters of each tissue type were observed along with a clear developmental gradient of tuber sizes (Fig. 5A). This was not observed, however, in the PCA of ElevT, where the clusters of IMT and MAT are more overlapping (Fig. 5B, red box). This suggests that ElevT has a differential impact on development and tuber identity compared to AmbT with regard to gene expression.

Differential expression analysis was then completed to determine genes with significant increases or decreases in expression across treatments, tissues, and time points using *DESeq2* (Love et al. 2014). Genes with adjusted *P*-value of <0.05 using a Benjamini–Hochberg correction method were considered differentially expressed. In leaves, there were a total of 1,078 differentially expressed genes (DEGs) between ElevT and AmbT across all time points, with 15, 615, 146, and 302 DEGs at 30-, 60-, 90-, and 120-d comparisons, respectively (Supplementary Table S4, A to D). In TI, there were a total of 26, 321, 302, and 6,257 DEGs in the 30-, 60-, 90-, and 120-d comparisons, respectively (Supplementary Table S5, A to D).

Gene ontology (GO) association of biological processes was completed on DEGs in ElevT for TI. These processes were identified for both DEGs with increased and decreased expression at each time point (Fig. 5, C and D). DEGs with increased expression in TI in ElevT were involved in response to heat, regulation of circadian rhythm, and calmodulin binding (Table 1). DEGs with decreased expression in TI in ElevT were involved in the regulation of flower development, lateral root formation, and response to cold (Table 2). More so, a clear overlap between phytohormone and transcriptome expression was seen in processes involving ABA and auxin as shown in Fig. 5, C and D.

**Table 1.** Subset of DEGs with increased expression found in TI and their associated GO term

Time point	GO term	ATL transcript ID	ATL functional annotation
30 d	Heat acclimation	Soltu.Atl_v3.12_4G008690.1	Co-chaperone GrpE family protein
	Calmodulin binding	Soltu.Atl_v3.10_3G006070.1	Lipid transfer protein
	Vegetative to reproductive phase transition of meristem	Soltu.Atl_v3.06_2G022450.2	Ubiquitin-conjugating enzyme
60 d	Response to abscisic acid	Soltu.Atl_v3.S043390.1	Transmembrane amino acid transporter family protein
	Regulation of circadian rhythm	Soltu.Atl_v3.01_1G022500.4	Homeodomain-like superfamily protein
	Photoperiodism, flowering	Soltu.Atl_v3.04_3G013030.1	GIGANTEA protein (GI)
90 d	Response to heat	Soltu.Atl_v3.12_4G003540.1	Related to *Apetala-2* (AP2)
	Adventitious development	Soltu.Atl_v3.09_1G015110.1	Cytochrome P450, family 83, subfamily B, polypeptide
	Response to sucrose	Soltu.Atl_v3.S027640.4	Alcohol dehydrogenase
120 d	Response to abscisic acid	Soltu.Atl_v3.09_4G023670.3	Mitochondrial transcription termination factor family protein
	Response to heat	Soltu.Atl_v3.12_2G026260.1	temperature-induced lipocalin
	Plant organ morphogenesis	Soltu.Atl_v3.S105180.1	AINTEGUMENTA-like protein

The GO term, Atlantic genome (ATL_v3) transcript identifier, and functional annotation are provided.

**Table 2.** Subset of DEGs with decreased expression found in TI and their associated GO term

Time point	GO term	ATL transcript ID	ATL functional annotation
30d	Lateral root formation	Soltu.Atl_v3.08_0G002640.2	Acyl transferase/acyl hydrolase/lysophospholipase superfamily protein
	JA-mediated signaling pathway	Soltu.Atl_v3.10_0G019400.2	Ubiquitin-specific protease
	Regulation of timing of transition from vegetative to reproductive phase	Soltu.Atl_v3.03_4G023830.1	Transducin family protein/WD-40 repeat family protein
60d	Ethylene-activated signaling pathway	Soltu.Atl_v3.01_0G011970.1	Ethylene-responsive element-binding factor
	Response to abscisic acid	Soltu.Atl_v3.04_3G011570.1	Alcohol dehydrogenase
	Plant-type hypersensitive response	Soltu.Atl_v3.02_4G008170.1	PATATIN-like protein
90d	Response to auxin	Soltu.Atl_v3.07_4G010580.1	SAUR-like auxin-responsive protein family
	Positive regulation of flower development	Soltu.Atl_v3.05_4G021720.1	PEBP family protein
	Response to cold	Soltu.Atl_v3.03_4G009740.1	Dehydration response element B1A
120d	Auxin biosynthetic process	Soltu.Atl_v3.06_4G001800.1	Flavin-binding monooxygenase family protein
	Response to abscisic acid	Soltu.Atl_v3.07_4G009150.1	Membrane bound O-acyl transferase family protein
	Abscisic acid catabolic process	Soltu.Atl_v3.08_1G000950.1	Cytochrome P450, family 707, subfamily A, polypeptide

The GO term, Atlantic genome (ATL_v3) transcript identifier, and functional annotation are provided.

As no MATs were obtained at the 90-d time point in plants grown in ElevT, we took a closer look at gene expression of TI at 60 d to gain insights into how heat stress might impact tuber filling. In TI at 60 d (Supplementary Table S5B), there were over 20 distinct genes related to heat shock proteins that were differentially expressed. It was also found that *GIGANTEA* (Soltu.Atl_v3.04_3G013030.1) had a significant increase in expression as ElevT. This is significant because *GIGANTEA* acts as upstream regulator of *StCOL1*, which represses tuberization. At the 90-d time point, we saw several genes containing domains related to *Apetela 2*, which is the same domain contained by *RELATED TO APETALA2 1* (*StRAP1*), which has been shown to decrease tuber yield (Dutt et al. 2017). We also detected 4 sucrose synthase genes (Soltu.Atl_v3.09_1G016720.5, Soltu.Atl_v3.12_1G010580.1, Soltu.Atl_v3.12_2G025220.1, and Soltu.Atl_v3.12_3G024000.1), 4 starch branching enzymes (Soltu.Atl_v3.04_1G022720.3, Soltu.Atl_v3.04_1G022720.3, Soltu.Atl_v3.04_2G020920.1, and Soltu.Atl_v3.04_3G022400.1) and a starch synthase gene (Soltu.Atl_v3.02_2G032060.2) that had significantly lower expression in TIs in ElevT at 90 d (Supplementary Table S5C).

### Expression patterns of known regulators in tuberization signaling

**Figure 6.**
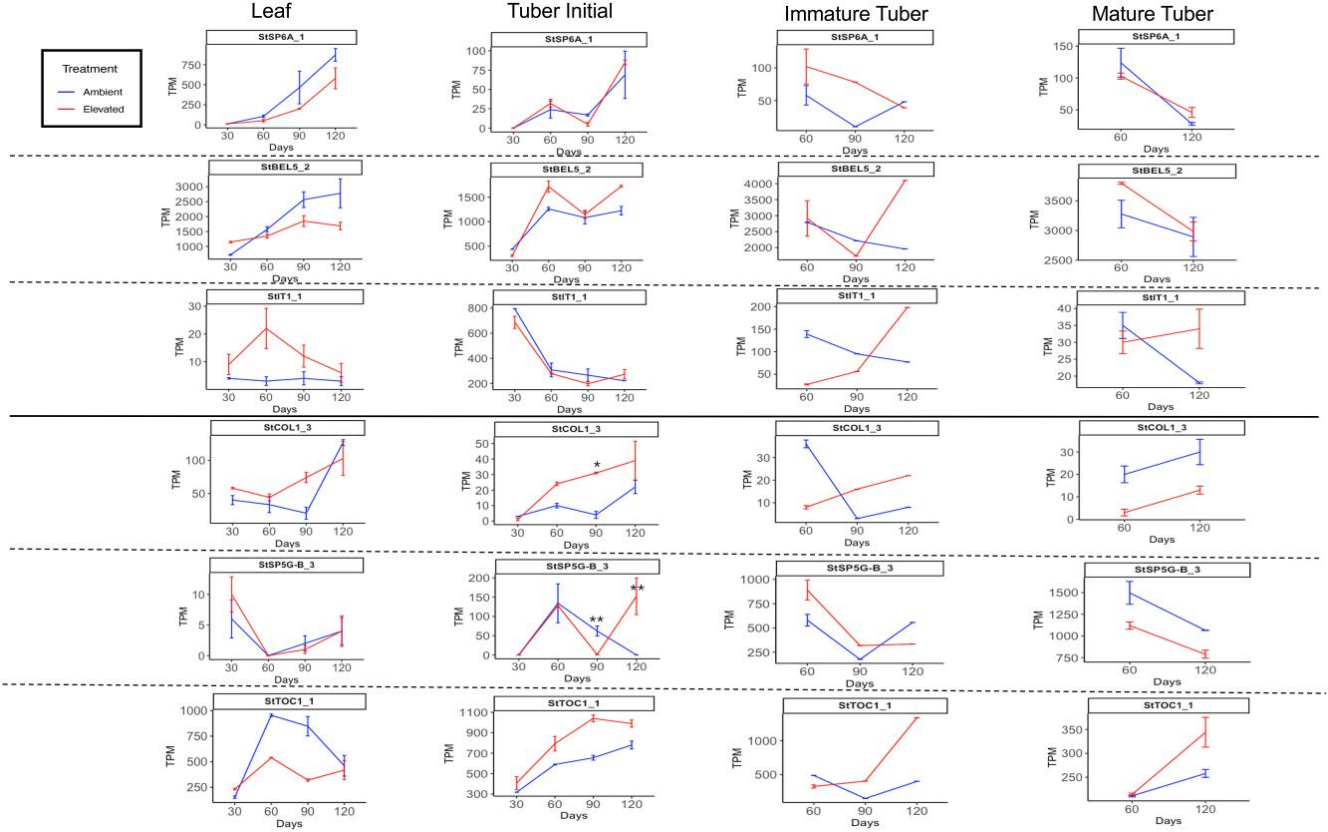
Transcript abundance (TPM) of known tuberization genes in each tissue type. **A to C)** Tuberization-promoting genes (**A**, *StSP6A*; **B**, *StBEL5*; and **C**, *StIT1*). **D to F)** Tuberization inhibitor genes (**D**, *StCOL1*; **E**, *StSP5G-B*; and **F**, *StTOC1*). TPM values were determined from *Salmon* (Patro et al. 2017) and averaged per biological replicate (*n* = 1 or 2). Blue lines represent plants grown in AmbTs while red lines represent plants grown in projected ElevTs. No ElevT MATs were collected at 90 d, so no data are shown for that point. Error bars represent the standard error. Significance was determined by the Wald test using *DESeq2* (Love et al. 2014). *P*-values were adjusted for false discovery rate using a Benjamini–Hochberg correction method (*P < 0.10; **P < 0.05).

We then investigated the gene expression patterns of key genes and known regulators involved in tuberization signaling in potato. For each key gene of interest, we provide expression information for 1 syntelog in this section. Syntelogs graphed in Fig. 6 in the manuscript represent syntelog(s) found to be differentially expressed or those with the highest transcript abundance. Supplementary Figures S5 to S11 provide expression data for all syntelogs associated with each gene of interest, and individual results from our syntelog analysis are in Supplementary Table S6. The genes of interest observed that induce tuberization include *StSP6A* (Soltu.Atl_v3.05_1G024440.1), *StBEL5* (Soltu.Atl_v3.06_3G019920.1), and *StIT1* (Soltu.Atl_v3.06_2G021580.1) (Fig. 6, A to C; Supplementary Figs. S5 to S7). Expression of key genes known to inhibit tuberization were also analyzed, including *StCOL1* (Soltu.Atl_v3.02_1G027420.1), both *StSP5G-A* (Soltu.Atl_v3.05_3G022510.1) and *StSP5G-B* (Soltu.Atl_v3.05_3G022530.1), and *StTOC1* (Soltu.Atl_v3.06_4G015370.2) (Fig. 6, D to F; Supplementary Figs. S8 to 11).

**Figure 7.**
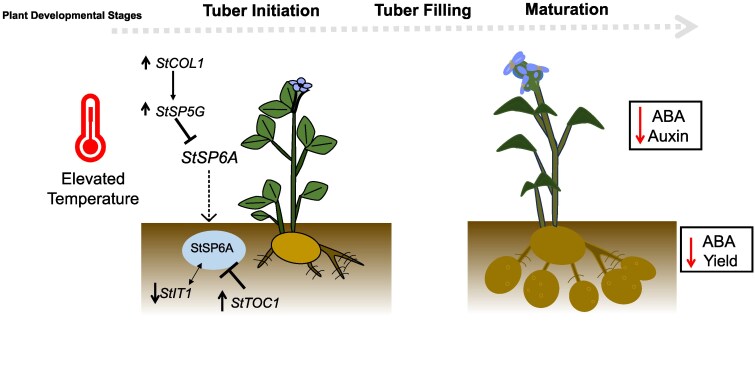
Model for known and potential changes in gene expression and hormone levels under ElevT. Dashed lines with arrows indicate movement, and small arrows indicate increases or decreases in expression under ElevT. Lines with closed ends indicate inhibition while double-arrowed lines between genes/proteins indicate interaction. Enclosed texts are metabolite and plant physiology-related parameters corresponding to above and belowground changes. Red arrows indicate change in physiology or phytohormone variables. Blue oval represents protein version of a *StSP6A*.

*StSP6A* expression in leaves was consistent with its known gradual expression pattern (Park et al. 2022) among syntelogs and tissues in both treatments (Fig. 7A; Supplementary Fig. S5). *StBEL5*, which is known to be a transcriptional activator that targets *StSP6A*, was also not differentially expressed at any time point (Sharma et al. 2016). Additionally, the expression pattern of *StBEL5* syntelogs was observed to be almost identical with exception of syntelog *StBEL5_3* (Fig. 6B; Supplementary Fig. S6).

In AmbT, *StIT1* expression was highest in TI at 30 d and then decreased by 61% by 60 d and eventually decreased to its lowest expression in AmbT at 120 time point (Fig. 6C). Due to the early role of *StIT1* in promoting tuberization of stolons, we would expect expression of this gene to be lower at later stages of tuber development (IMT and MAT), which is what was observed (Fig. 6C). *StIT1* had relatively lower abundance in ElevT in TI tissue but was not identified as differentially expressed due to ElevT (Supplementary Fig. S7).

In terms of repressors of tuber formation, 1 *StCOL1* syntelog was observed to have increased expression at 90 d in TI (Fig. 6D; *P* < 0.08). When examining *StSP5G*, *StSPG-A* and *StSP5G-B* had similar expression trends in tubers; however, none of the *StSPG-A* syntelogs were differentially expressed. In contrast, some syntelogs of *StSP5G-B* in TI were differentially expressed at both 90 and 120 d, while all syntelogs were observed to have increased in expression at 120 d in ElevT (Fig. 6E; Supplementary Figs. S9 and 10). *StSP5G* has been previously identified as a downstream target of *StCOL1* and to be differentially expressed by ElevT (Hancock et al. 2014). *StTOC1* showed increased expression in all tuber classes under ElevT but was not significantly differentially expressed (Fig. 6F; Supplementary Fig. S11). In leaves, we saw nonsignificant reduction in the expression of *StTOC1* at all time points except at 30 d (Fig. 6F).

## Discussion

### Tuber yield loss under heat stress is not caused by a decrease in photosynthetic parameters

In this study, we investigated the mechanisms of yield loss that is observed when potato plants are exposed to chronic ElevT. From leaf physiology measurements, we found that *A* and *g_s_* were not significantly decreased in ElevT compared to AmbT at every time point other than 60 d when these plants were found to have drier soil than in AmbT (Fig. 1). Upon experiencing water deficit, plants produce signals to close their stomatal pores, thus reducing transpiration and conserving water, which significantly reduces gas exchange of leaves (Galmés et al. 2013). As such, the significantly lowered rates of *A* and *g_s_* at the 60-d time point are most likely due to insufficient watering. Our results also suggest that future project ElevTs for potato do not significantly impact leaf senescence (as seen through changes in *F_v_*/*F_m_*) or chlorophyll content when water availability is not a concern (Supplementary Figs. S1 and S2). These results are consistent with other studies that found no significant or positive effects of high temperatures on photosynthetic rates and chlorophyll content in potato (Hancock et al. 2014; Park et al. 2022).

Despite lack of significant effects of ElevT on leaf physiology in our experiment, we still observed a significant decrease in tuber number and yield from ElevT plants (Figs. 2 and 3; Supplementary Table S1). This implies that ElevTs affect the aboveground and belowground components of potato plants differently. Moreover, many studies indicate significant increases in aboveground biomass of potato plants exposed to high temperatures (Timlin et al. 2006; Tang et al. 2018). Although there were no significant differences in aboveground biomass observed in this study, measurements increased slightly under ElevT (Fig. 3, C to E), consistent with findings in the literature. Thus, results from our study suggest a reduction in photoassimilate production is not a driver of yield loss under heat stress.

### Changes in phytohormone content in potato under heat stress may impact tuberization

Internal signaling in potato plants is crucial for the formation of tubers and maintenance of sink strength, both through genetic regulation and through hormone signaling (Dutt et al. 2017). In the present study, our phytohormone analyses revealed several changes in phytohormone content. This included significantly lower levels of ABA active form across all tissue types under ElevT (Fig. 4C). Besides its role in stress response, ABA contributes to tuber development in *S. tuberosum* through antagonistic action on GA (Li et al. 2020; Chen et al. 2022). GA is an important inhibitor of tuberization through inducing stolon elongation, which in turn inhibits tuber induction by repressing radial growth of stolons into tubers (Xu et al. 1998). ABA is also known to regulate dormancy of potato tubers, with lower levels generally associated with shorter dormancy periods (Wang et al. 2020). In this regard, the decreased ABA levels observed in tubers under ElevTs could explain the reports of heat sprouts and early dormancy breaks seen in potatoes grown under high temperature in other studies (Zhang et al. 2021). Recent work showed that *StABI5-like 1* (*StABL1*), a transcription factor central to ABA signaling, is a binding partner of *FT-like* genes *StSP6A* and *StSP3D*. When StABL1 interacted with StSP6A and StSP3D, it was shown that an alternative tuberigen activation complex is formed and promotes tuberization (Jing et al. 2022). Overexpression of this *StABL1* gene resulted in earlier flowering, tuberization and overall shorter plant life cycle. Coupled with the finding of ABA-associated DEGs from our TI tissue (Fig. 5, C and D), these results indicate that further work is needed to understand the role of ABA signaling in tuber development under higher temperatures.

### Chronic ElevTs inhibit tuber filling, but not initiation

Tuber production of a potato plant consists of 3 main separate processes: tuber formation/initiation, tuber filling, and tuber maturation stage (Liu et al. 2020). Quantification of the number of TI and IMT produced under ElevT showed no significant change compared to AmbT (Fig. 2; Supplementary Table S1). However, we observed an increased average number of TI at 30 d in ElevT compared to AmbT (Fig. 2). These results suggest that tuber initiation is not significantly inhibited under chronic ElevTs. Nevertheless, we did observe a significant decrease in MAT yield collected from ElevT plants (Fig. 2; Supplementary Table S1). This implies a disruption in the normal developmental process under ElevT, hindering tubers from reaching maturity or filling adequately. Past studies with acute ElevT treatments have shown it negatively impacts potato yield by reducing amount of fully matured potato tubers (Kim and Lee 2019).

Additionally, there were observable differences between RNA-seq clustering between treatments. Plants grown in ElevT conditions showed stronger clustering of MAT and IMT libraries compared to MAT and IMT samples from plants grown in AmbT conditions (Fig. 5, A and B). This indicates that IMT from plants grown in ElevT conditions exhibit more similar transcript expression patterns to MAT than when grown in AmbT. This aligns with previous reports that identified an increase in physiological age of tubers because of ElevTs (Weirsema 1985). Therefore, chronic heat stress increases the developmental age of tubers, which in turn decreases the potato in-season growing time and its postharvest tuber dormancy time (Weirsema 1985). This implies that heat stress caused by future climate change likely disrupts normal signaling patterns that occur between tuber initiation and maturation, impacting tuber development and ultimately yield.

### Changes in expression of tuberization genes were observed in ElevT

There has been considerable work in recent years on understanding the effects of ElevT on the tuberigen *StSP6A* expression and its role in tuberization (Lehretz et al. 2019; Park et al. 2022). A previous study observing transgenic *StSP6A* overexpressor lines concluded that *StSP6A* likely controls tuber initiation, but not tuber filling as yield was still significantly decreased in *StSP6A* overexpressor lines under high temperatures (Park et al. 2022). In our current study, there were no significant differences with expression of *StSP6A* under ElevT (Fig. 6A). We also found the tuberigen repressor, *StSP5G-A*, had preferential expression in leaves as well as relatively higher expression in leaf tissue compared to its paralogous *StSP5G-B*; however, *StSP5G-B* had significantly increased expression under ElevT at 120 d in TI (Supplementary Figs. S9 and S10). These results align with past studies that have observed higher expression of *StSP5G* under ElevT in leaves (Hancock et al. 2014), while we also observed them to be DEGs in TI.

Moreover, *StCOL1*, which is known to induce expression of *StSP5G*, was observed to have increased expression in TI under ElevT at 90 d (Fig. 6D). *StCOL1* is a *CONSTANS-like* gene part of a well-described *CONSTANS/FT* (*CO/FT*) module that is central for sensing photoperiod and regulating developmental processes within Arabidopsis (*Arabidopsis thaliana*) and more recently in other plants (Turck et al. 2008). In AmbT, we see similar expression patterns between FT-like *StSP6A* and *StCOL1* in leaves, which is crucial for proper photoperiod sensing (Supplementary Figs. S5 and S8; Fig. 6). On the other hand, expression of *StCOL1* in leaves grown in ElevT conditions shows a distinct pattern to *StSP6A* across sampled time points (Supplementary Figs. S5 and S8; Fig. 6). Taken together, *StCOL1* may be involved both in misperception of environmental signals at ElevT through improper coordination of the *StCOL1/StSP6A* module in leaves, as well as contributing to the reduction of tuber yield through induction of tuber repressors (*StS5PG*) at later stages of the plant life cycle (90 d) in ElevT conditions. Although our results provide insight into heat-driven misregulation of these key repressors, the downstream effects of these disruptions on carbohydrate transport and storage in tubers remain unclear and warrant further investigation.

An additional inhibitor of *StSP6A*, *StTOC1*, has been recently found to have increased expression in ElevT through in vitro tuberization studies (Morris et al. 2019). From our RNA-seq data, we found no significant difference in expression of *StTOC1* albeit we observed increased expression over time in all tuber tissue classes under ElevT compared to AmbT (Fig. 6F). Within IMT and MAT, we also observe opposite expression patterns of *StTOC1* with *StSP6A*, indicating *StTOC1* may inhibit transcription of *StSP6A* in IMT and MAT near the end of the growing season. These data are consistent with previous studies that have found increases in expression of *StTOC1* in tubers under higher temperatures (Hancock et al. 2014). This opens an avenue for research to determine whether the persistence of high *StTOC1* expression in heat-stressed tubers could alter the plant's ability to prioritize tuber filling over vegetative growth.

One tuberization pathway that remains unexplored under ElevT is through *IDENTITY OF TUBER* (*StIT1*). *StIT1* is a gene found to both interact with *StSP6A* and induce tuberization under non-inductive conditions (Tang et al. 2022). From our data, we observed nonsignificant difference in expression due to ElevT although we observed consistently less expression of *StIT1* in all tuber tissues under ElevT except at the 120-d time point. Notably, it is at the 120-d time point when we see a recovery of tuber yield, as at 90 d, we saw no MATs had developed in our ElevT treatment. The role of *StIT1* in regulating tuberization under chronic heat stress remains poorly understood, and further exploration of *StIT1* interactions with other tuberization regulators, such as *StSP6A*, could shed light on whether this gene promotes heat resilience in potatoes.

Taken together, our results build on previous studies by providing insights into how *StSP6A* is affected by the *StSP5G*-*StCOL1* pathway under chronic heat stress. Notably, the key tuber induction genes, including *StSP6A* and *StBEL5*, did not show significant changes in expression under ElevTs, suggesting that their role in tuber initiation may remain intact even under heat stress. In contrast, we observed that several repressor genes—such as *StSP5G*, *StCOL1*, and *StTOC1*—were more responsive to ElevTs, exhibiting increased expression in tuber tissues. This differential sensitivity between inducers and repressors points to a potential shift in regulatory control, where the overexpression of repressors may play a more dominant role in limiting tuber filling rather than initiation.

## Conclusion

The expected rise in global temperatures in coming decades poses an impending threat to the production of many staple crops, especially ones that are as susceptible to heat stress such as *S. tuberosum*. Understanding the effects of abiotic stress on crop plants is thus a crucial goal for coping with climate change. Here, we take a whole-plant approach to investigate the mechanisms of potato yield loss under heat stress using a realistic projection of future climate for major potato-growing regions. Leaf-level physiology, endogenous phytohormone levels, and yield of potato plants grown under chronic ElevT were measured. Leaf gas exchange parameters (*A* and *g_s_*) were not significantly negatively impacted by ElevT nor were leaf chlorophyll content or rates of leaf senescence. We observed a significant decrease in leaf ABA and auxin levels in leaves and tuber tissues under ElevT conditions, which indicates a possible role of these hormones in tuberization signaling in potato grown in ElevT. ElevT conditions lead to a reduction in the average number of tubers per plant at all time points except at 30 d. From this, we hypothesize that chronic ElevT is impacting tuber yield by inhibiting proper tuber filling and not by inhibiting tuber initiation. We also characterized the expression of genes under chronic ElevT that are known contributors to tuberization. Results from this analysis contribute to the intricate understanding of the genetic regulation of tuberization under ElevTs in potato plants, highlighting the potential role of the *StCOL1-StSPGB* pathway as one target for generating heat-tolerant potato cultivars (Fig. 7). Other potential candidate genes to be explored are *StTOC1* and *StIT1*, offering different genetic pathways to sustain and enhance potato yield in the face of global warming and ElevTs.

## Materials and methods

### Growth chamber experimental design

Potato plants were grown from seed tubers of the chip-processing cultivar Manistee in a controlled growth chamber experiment. In 2021, seed tubers were obtained from Michigan State University, courtesy of Dr. Dave Douches, and stored at room temperature for 3 wk to break dormancy. Sprouted seed tubers were cut into halves and planted in 14.2-L pots with Promix BX soil. Plants were grown using 60% relative humidity in controlled growth chambers (Conviron Adaptis; Controlled Environments, Inc) under either AmbT or ElevT conditions. AmbT conditions represented the average temperature (minimum and maximum) from 1980 to 2000 (the established historical control climate period) while ElevT conditions were those projected for the mid-21st century (2040 to 2060) in a major potato production region in the United States (Eau Claire, MI) (Leisner et al. 2017) (for full description of temperature values, see Supplementary Table S7). Minimum and maximum temperatures ranged from 11.1 to 28.8 °C in AmbT and 13.8 to 33 °C in ElevT.

**Figure 8.**
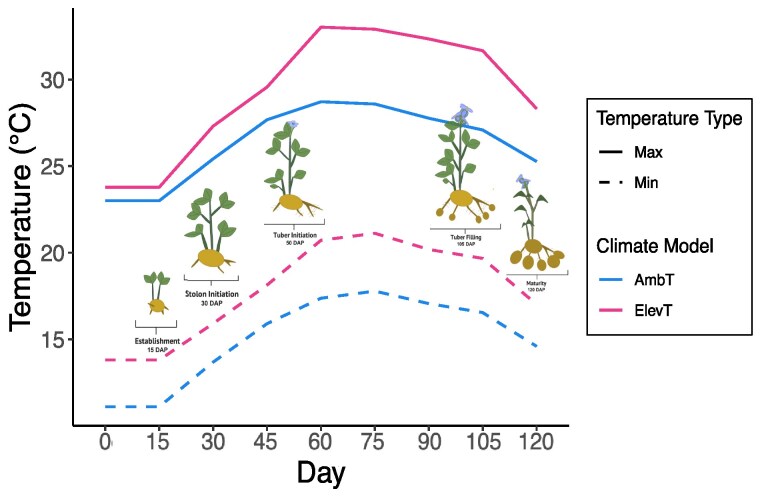
Maximum and minimum temperatures for the historical climate control period (1980 to 2000) and projected future temperature for the mid-century (2040 to 2060) using the CRCM_cgcm3 climate model with plant development stages superimposed on the temperature values. Temperatures are given for the 120-d growing period for potato. CRCM_cgcm3 data obtained from Leisner et al. (2017).

Future projections of surface minimum and maximum temperature for the ElevT growth conditions were obtained from an ensemble of a dynamically downscaled climate simulations produced by the North American Regional Climate Change Assessment Program (Mearns et al. 2012). The climate projections are developed from a combination of regional climate model (RCM) and global climate model (GCM) simulations. The RCM/GCM combination used for this experiment was the Canadian Regional Climate Model (CRCM)/Canadian Global Climate Model version 3 (CGCM3), thereafter referred to as CRCM_cgcm3. The climate model used in this experiment (CRCM_cgcm3) projects that by the mid-21st century, the largest change in maximum and minimum temperature in Michigan will occur during tuber development (initiation and filling) (Fig. 8) and has previously been observed to impact tuber yield (for detailed methodology on the GCM downscaling, see Leisner et al. 2017).

Minimum and maximum temperatures along with photoperiod changed every 2 wk to mimic seasonal fluctuations during a normal potato growing season (mid-May to mid-September) (Fig. 8; Supplementary Table S7). The light intensity in the growth chambers was measured at the top of the canopy using a LI-180 spectrometer (LICOR Biosciences, Lincoln, NE, USA) and ranged from 300 to 500 *μ*mol m^−2^ s^−1^, and the height of the light rack was adjusted every 2 wk to maintain these light levels as plants grew. Plants were watered twice a week with 1 L of water per plant until 60 d, after which they were watered 2 to 3 times a week to maintain similar soil moisture. Each chamber was fertilized with ∼14-g Jack's Classic All Purpose 20-20-20 Fertilizer diluted in 7 L of water once a week once sprouts emerged, about 2 wk after planting (JR Peters Inc., Allentown, PA, USA). The plants were rotated twice a week across chambers of the same treatment to minimize chamber effects. Sampling was done on 2 to 4 plants of each treatment every 30 d after planting until maturity (120 d). After sampling, the plants were removed from the chamber. Samples taken from plants within the same chamber at the same time point were pooled together as technical replicates, and each chamber was used as a biological replicate (*n* = 2).

### Photosynthetic gas exchange, chlorophyll fluorescence, and chlorophyll content measurements

Midday gas exchange measurements of 1 leaf per plant were recorded in situ between 11:00 and 13:00 to determine photosynthetic CO_2_ assimilation (*A*) and stomatal conductance (*g_s_*) using a LI-6800 portable photosynthesis system (LICOR Biosciences, Lincoln, NE, USA). Leaf temperatures were set to reflect chamber settings (16.5 and 18.7 °C at 30 d, 20.1 and 23.8 °C at 60 d, 19.9 and 23.4 °C at 90 d, and 17.7 and 20.3 °C at 120 d for AmbT and ElevT temperatures, respectively). Relative humidity, CO_2_ concentration, and light intensity levels were also set to mimic conditions within the growth chambers. Following gas exchange measurements, 2 fresh leaves from each sampled plant were collected and 4 6-mm diameter leaf discs were taken, placed adaxial side up on 3 mm MES buffer. Maximum PSII quantum efficiency of the leaf discs was measured in terms of *F_v_*/*F_m_*, which is variable fluorescence (*F_v_*) divided by the maximum fluorescence (*F_m_*), at 24, 48, 72, and 96 h after dark exposure as described by Zwack et al. (2016). Changes in *F_v_*/*F_m_* were taken to measure the rate of leaf senescence. After 4 d, each leaf disc was then transferred to 500 *µ*L of 100% methanol overnight, and a UV/Vis spectrophotometer was used to measure the total chlorophyll of the methanol extract for each leaf disc as described by Zwack et al. (2016) (Beckman Coulter, USA).

### Biomass and yield measurements

Aboveground biomass and yield measurements were taken at 120 d for 4 plants per chamber. Plant height was taken by measuring the tallest point of each plant. The entire aboveground plant was cut off, and FW was recorded before placing the plants in a 60 °C dryer for 2 wk to measure DW. Tubers were collected fresh, counted per chamber, and categorized into 3 different developmental classes based on weight: TIs (<0.6 g), IMTs (0.6 to 5 g), and MATs (>5 g). Examples of each tuber size class are given in Supplementary Fig. S3. For tuber count and weight measurements at each sampling point, 2 or 4 individual plants were sampled from each condition. Two plants were sampled at 30 and 90 d, while 4 plants were sampled at 60 and 120 d. The final tuber yield per chamber (at 120 d) was taken by weighing all tubers with a mass greater than 0.6 g (TIs were excluded from the final yield). Values were averaged across chambers (*n* = 2).

### Phytohormone extraction and analysis

Phytohormone analysis was done on leaf samples at 90 and 120 d. Tissue samples were collected from 1 leaf per plant (4 plants total) and immediately flash frozen in liquid nitrogen. Tubers were collected and separated into size classes before flash freezing. Pooled tissue from 90- and 120-d samples were ground into a fine powder and stored at −80 °C. Between 10 and 20 mg of frozen ground tissue powder were placed into microtubes and lyophilized overnight in a FreeZone 1 freeze–dry system at −50 °C (LabConco, Kansas City, MO, USA). Phytohormone determination was done in the Laboratory of Hormonal Regulations in Plants, Institute of Experimental Botany of the Czech Academy of Sciences as previously described in Prerostova et al. (2021). Homogenized samples (ca. 1.5 to 2 mg DW) were extracted with 100-*µ*L 1 m formic acid solution. Mixtures of stable isotope-labeled phytohormone standards were added at 1 pmol per sample. The extracts were centrifuged at 30,000 × *g* for 25 min at 4 °C. The supernatants were applied to SPE Oasis HLB 96-well column plates (10 mg/well; Waters, Milford, MA, USA) conditioned with 100-*µ*L acetonitrile and 100-*µ*L 1 m formic acid using Pressure+ 96 Manifold (Biotage, Uppsala, Sweden). After washing wells 3 times with 100-*µ*L water, the samples were eluted with 100-*µ*L 50% acetonitrile in water. An aliquot of the extract was analyzed on a LC/MS system consisting of UHPLC 1290 Infinity II (Agilent, Santa Clara, CA, USA) coupled to 6495 Triple Quadrupole Mass Spectrometer (Agilent, Santa Clara, CA, USA), operating in MRM mode, with quantification by the isotope dilution method.

Internal standards used for the phytohormone analysis were as follows: ^13^C_6_-IAA (Cambridge Isotope Laboratories, Tewksbury, MA, USA); ^2^H_4_-SA (Sigma-Aldrich, St. Louis, MO, USA); ^2^H_3_-PA and ^2^H_3_-DPA (both from NRC-PBI, Saskatoon, Canada); and ^2^H_5_-tZ, ^2^H5-tZR, ^2^H5-tZ7G, ^2^H5-tZ9G, ^2^H5-tZOG, ^2^H_5_-tZROG, ^2^H_5_-tZRMP, ^15^N_4_-cZ, ^2^H_3_-DZ, ^2^H_3_-DZR, ^2^H_3_-DZ9G, ^2^H_3_-DZRMP, ^2^H_6_-iP, ^2^H_6_-iPR, ^2^H_6_-iP7G, ^2^H_6_-iP9G, ^2^H_6_-iPRMP, (^2^H_5_)(^15^N_1_)-IAA-Asp, (^2^H_5_)(^15^N_1_)-IAA-Glu, (^2^H_5_)(^15^N_1_)-IAM, ^2^H_6_-ABA, ^2^H_5_-JA, ^2^H_2_-GA1, ^2^H_2_-GA4, ^2^H_2_-GA8, ^2^H_2_-GA12, and ^2^H_2_-GA19 (all from Olchemim Ltd., Olomouc, Czech Republic).

Data acquisition and processing were performed with Mass Hunter software B.08 (Agilent, Santa Clara, CA, USA). Three technical replicates per sample were averaged together, and technical replicates with a relative standard deviation higher than 30% were excluded. Biological replicates (*n* = 2) were then averaged together and analyzed using an ANOVA.

### RNA extraction and sequencing

Total RNA was extracted from the same frozen ground tissue used for the phytohormone analysis. RNA from leaf and tuber tissue collected at each time point was done using the Spectrum Plant Total RNA Extraction Kit (Sigma-Aldrich, St. Louis, MO, USA). The RNA was treated with Turbo DNA-free kit, and concentration and integrity were quantified using a NanoDrop Microvolume UV-Vis Spectrophotometer (Thermo Scientific, Wilmington, DE, USA) and 2100 Bioanalyzer (Agilent, Santa Clara, CA, USA). Library preparation and sequencing were performed by Novogene (Novogene Corporation, Inc., Sacramento, CA, USA). Libraries were sequencing using the Illumina NovaSeq 6000 platform producing 150-bp paired-end reads. A total of 49 libraries were sequenced, with an average of 44,601,280 total reads per library (Supplementary Table S3).

### Differential gene expression analysis

RNA reads from sequencing were quality trimmed (*P* > 20), and adapters were removed using *fastp* (v0.23; Chen 2023), and quality checks were completed using *fastQC* (v0.11.9; Chen et al. 2018). The reads were aligned to the tetraploid Atlantic potato version 3 (ATL_v3) genome (Hoopes, Meng, et al. 2022; Hoopes, Zarka, et al. 2022) from SpudDB using *HISAT2* (v2.2.1; Kim et al. 2019; downloaded August 2023 from http://spuddb.uga.edu). Gene count matrices were generated with *featurecounts* (v2.0.3; Liao et al. 2014) using the representative high confidence gene models of the ATL_v3 genome. The raw count matrices can be found in Supplementary Data Set S1 and served as input for the *DEseq2* (v1.38.3; Love et al. 2014) R package in which visualization, modeling, and differential syntelog-specific expression analysis of the libraries were completed. Genes with read counts of 0 across all libraries were removed from the analysis. *P*-values were adjusted for false discovery rate using a Benjamini–Hochberg correction method, and genes with *P*-adjusted values of <0.05 were considered as DEGs. Functional annotations were downloaded from SpudDB (Downloaded August 2023 from http://spuddb.uga.edu). To visualize variation in gene expression across different tissues and treatments, a PCA was done to reduce the dimensionality of the data to identify underlying patterns. Normalized gene counts generated from *DESeq2* were used as input for the PCA. PCA figures were generated using *ggplot* in R (version 4.3.1).

### GO association

GO association was done on DEGs from this experiment. GO terms were assigned to the ATL_v3 gene models by searching the protein sequences against the Arabidopsis proteome (The Arabidopsis Information Resource [TAIR] 10) using *DIAMOND* (v2.1.8l; Buchfink et al. 2015) with an *e*-value cutoff of 1e^−5^. The top match was used to transfer the GO annotation from the TAIR 10 Gene Ontology Annotations. The GO terms were then slimmed using the *map2slim* from the go-perl package (v0.15; https://metacpan.org/dist/go-perl) to generate the final set of GO slim terms. The file “ATL_v3.working_models.go_slim.obo.gz” generated from this research has been uploaded to the SpudDB database (http://spuddb.uga.edu/ATL_v3_download.shtml). Additional information regarding all GO terms was obtained from TAIR (https://www.arabidopsis.org/download/overview on March 2024) to generate a full list of associated GO terms with each DEGs for TI (Supplementary Data Set S2).

### Identifying genes of interest in tuber development in the Atlantic potato genome

*BLAST* (Altschul et al. 1990) was used to identify previously characterized tuberization genes from the double-monoploid (DM) reference genome (v6.1; Pham et al. 2020) in the recently published ATL_v3 genome (Hoopes, Meng, et al. 2022; Hoopes, Zarka, et al. 2022). SpudDB (http://spuddb.uga.edu/) was used to obtain nucleotide sequences of previously characterized genes in DM and then used as query sequences for a nucleotide *BLAST* (blastn) against the ATL nucleotide database within SpudDB. Additionally, *OrthoFinder* (v2.5.4; Emms and Kelly 2019) was used to confirm orthology between ATL genome genes of interest and DM genome genes of interest. Based on our findings, sequences in the ATL genome were labeled in accordance with their existing DM gene nomenclature, augmented with numeric suffixes denoting different gene loci (i.e. syntelogs). Syntelogs are homologous genes resulting from a whole-genome duplication event or speciation and are derived from the same ancestral genomic region and are expected to share similar functions. Information regarding the reference DM locus and the corresponding ATL syntelog loci can be found within Supplementary Data Set S3. Transcripts per million (TPM) values of the genes of interest were obtained from *Salmon* (v.1.9.0; Patro et al. 2017) using alignment mode quantification with the transcript sequences (cDNA) of the representative high confidence gene models set available for the ATL_v3 genome in SpudDB. From our tissue sampling, we then averaged TPM values for each syntelog for each tissue type at every time point (*n* = 2). Exceptions to the average TPM value are TIs at 30 d and IMTs at 90 and 120 d where only 1 biological replicate is available due to lack of sufficient tissue at these time points. Syntelogs graphed in Fig. 6 in the manuscript represent syntelog(s) found to be differentially expressed or those with the highest transcript abundance while all syntelogs from the genes described are in Supplementary Figs. S5 to S11. Visualization of the data in a Venn diagram was made using https://molbiotools.com/listcompare.php.

### Statistical analysis of physiology data

Assumptions of normality were validated using the Shapiro–Wilk test, and homogeneity of variance was checked using the Levene's test. *t*-tests, ANOVA, and Tukey post hoc tests on all physiology data were completed using the *stats* R package (R Core Team 2022).

### Accession numbers

Sequence data from this article can be found in the Small Read Archive under the BioProject ID PRJNA962840 (https://www.ncbi.nlm.nih.gov/sra). Accession identifiers as well as metadata information can be found in Supplementary Table S3. For genes studied in this article, gene identifiers for the Atlantic Genome available on Spuddb can be found in Supplementary Table S6.

## Supplementary Material

kiae664_Supplementary_Data

## Data Availability

Raw sequence data from this article can be found in the Small Read Archive under the BioProject ID PRJNA962840 (https://www.ncbi.nlm.nih.gov/sra). All other data underlying this article are available in the article and in its online supplementary material.

## Supplementary data

The following materials are available in the online version of this article.

**Supplementary Figure S1.** Measurement of maximum PSII efficiency (*F_v_*/*F_m_*) in plants grown in AmbT and ElevT.

**Supplementary Figure S2.** Leaf total chlorophyll content measured in AmbT and ElevT treatments.

**Supplementary Figure S3.** Example of tuber size classes for this experiment.

**Supplementary Figure S4.** PCA of all RNA-seq libraries.

**Supplementary Figure S5.** TPM values of the known tuberization-promoting gene *StSP6A* syntelogs in each tissue type over 120 d.

**Supplementary Figure S6.** TPM values of the known tuberization-promoting gene *StBEL5* syntelogs in each tissue type over 120 d.

**Supplementary Figure S7.** TPM values of the known tuberization-promoting gene *StIT1* syntelogs in each tissue type over 120 d.

**Supplementary Figure S8.** TPM values of the known tuberization inhibitor gene *StCOL1* syntelogs in each tissue type over 120 d.

**Supplementary Figure S9.** TPM values of the known tuberization inhibitor genes *StSP5G-A* syntelogs in each tissue type over 120 d.

**Supplementary Figure S10.** TPM values of the known tuberization inhibitor gene *StSP5G-B* syntelogs in each tissue type over 120 d.

**Supplementary Figure S11.** TPM values of the known tuberization inhibitor gene *StTOC1* syntelogs in each tissue type over 120 d.

**Supplementary Table S1.** Average number of tubers per plant for each time point and size class in AmbT and ElevT conditions.

**Supplementary Table S2A.** Endogenous hormone levels in pmol/g DW of all auxin metabolite.

**Supplementary Table S2B.** Endogenous hormone levels in pmol/g DW of all cytokinin metabolites.

**Supplementary Table S2C.** Endogenous hormone levels in pmol/g DW.

**Supplementary Table S2D.** Abbreviations and full names of all metabolites measured by LC/MS in the phytohormone analysis.

**Supplementary Table S3.** RNA-seq read counts and alignment quality for 49 sequenced libraries.

**Supplementary Table S4A.** Significant DEGs under ElevT in leaves at 30 d.

**Supplementary Table S4B.** Significant DEGs under ElevT in leaves at 60 d.

**Supplementary Table S4C.** Significant DEGs under ElevT in leaves at 90 d.

**Supplementary Table S4D.** Significant DEGs under ElevT in leaves at 120 d.

**Supplementary Table S5A.** Significant DEGs under ElevT in TI at 30 d.

**Supplementary Table S5B.** Significant DEGs under ElevT in TI at 60 d.

**Supplementary Table S5C.** Significant DEGs under ElevT in TI at 90 d.

**Supplementary Table S5D.** Significant DEGs under ElevT in TI at 120 d.

**Supplementary Table S6.** Gene names and transcript IDs of key tuberization genes.

**Supplementary Table S7.** Conviron Adaptis growth chamber settings.

**Supplementary Data Set S1.** RNA-seq raw count matrix generated from our sample libraries when aligned to the ATL_v3 genome. Columns represent sample library and rows represent genes.

**Supplementary Data Set S2.** Table containing all GO terms associated with each DEG in TI for each time point.

**Supplementary Data Set S3.** Syntelogs identified in this manuscript between DM and ATL genotypes with OrthoFinder.

## Dive Curated Terms

The following phenotypic, genotypic, and functional terms are of significance to the work described in this paper:

leaf AmiGo: PO:0025034

plant height Planteome: TO:0000207

TOC1 Gramene: AT5G61380
TOC1 Araport: AT5G61380
