# Supplementary material for: The physiological and molecular responses of potato tuberization to projected future elevated temperatures
Source: Plant Physiol. 2024 Dec 17;197(1):kiae664. doi: 10.1093/plphys/kiae664 (PMC11683837; doi:10.1093/plphys/kiae664)
Supplement: kiae664_Supplementary_Data [file kiae664_supplementary_data.zip › PP2024RA00921R1_Supplementary_Figures.pdf]

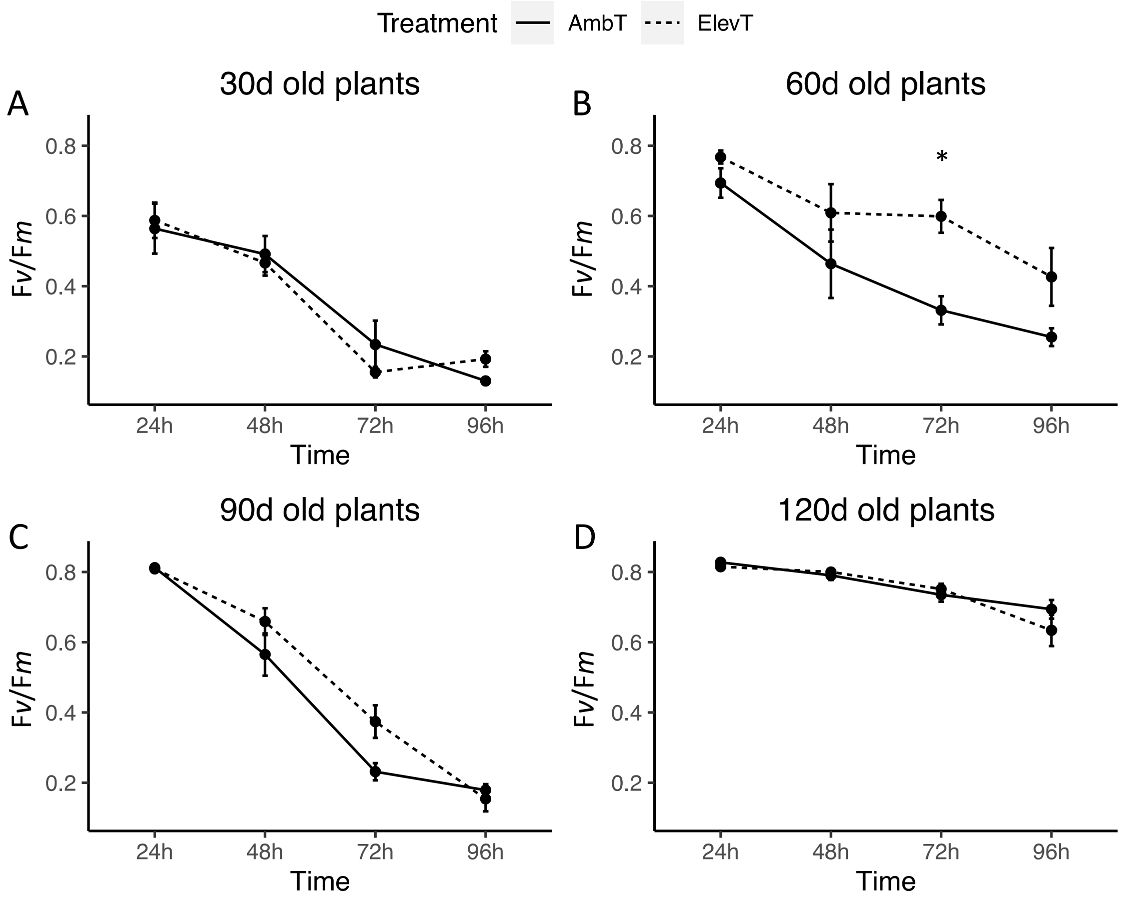

Supplementary Figure S1. Measurement of maximum PSII efficiency (F_v/F_m) in plants grown in ambient (AmbT) and elevated temperatures (ElevT). Measurements were taken on leaves over the course of 96h to represent leaf senescence. A) 30-day-old plants; B) 60-day-old plants; C) 90-day-old plants; D) 120-day-old plants. Error bars are standard error between biological replicates (n = 2). Asterisks indicate significant differences between AmbT and ElevT from pairwise T-tests at each hour of measurement (* = $p < 0.01$).

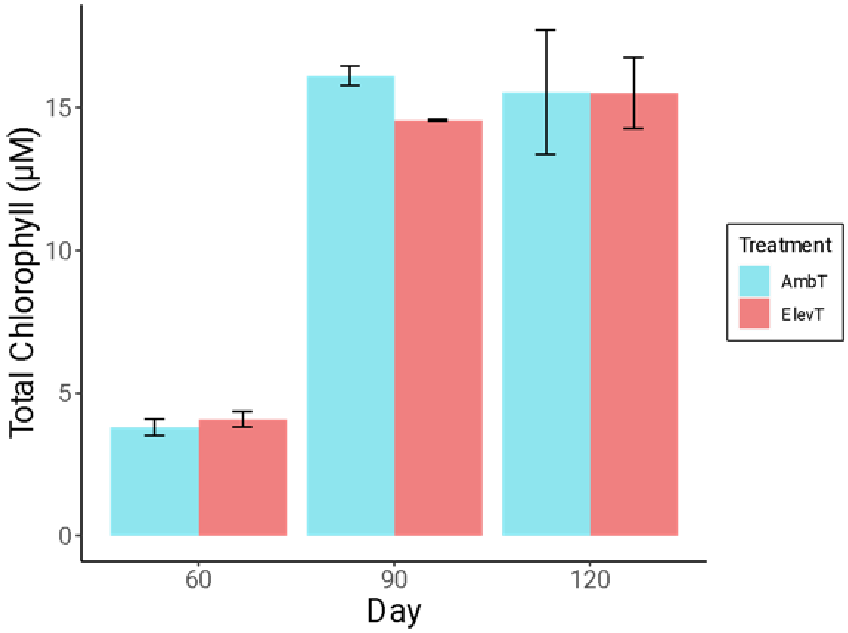

Supplementary Figure S2. Leaf total chlorophyll content measured in ambient (AmbT) and elevated temperature (ElevT) treatments. Total chlorophyll values are averaged per four 6mm leaf discs collected from 60-, 90-, and 120-d-old plants. Error bars indicate standard error between biological replicates ($n = 2$). Pairwise *T*-tests were completed between treatments at each time point. Significant differences between AmbT and ElevT were determined from pairwise *T*-tests at each hour of measurement ($p < 0.05$).

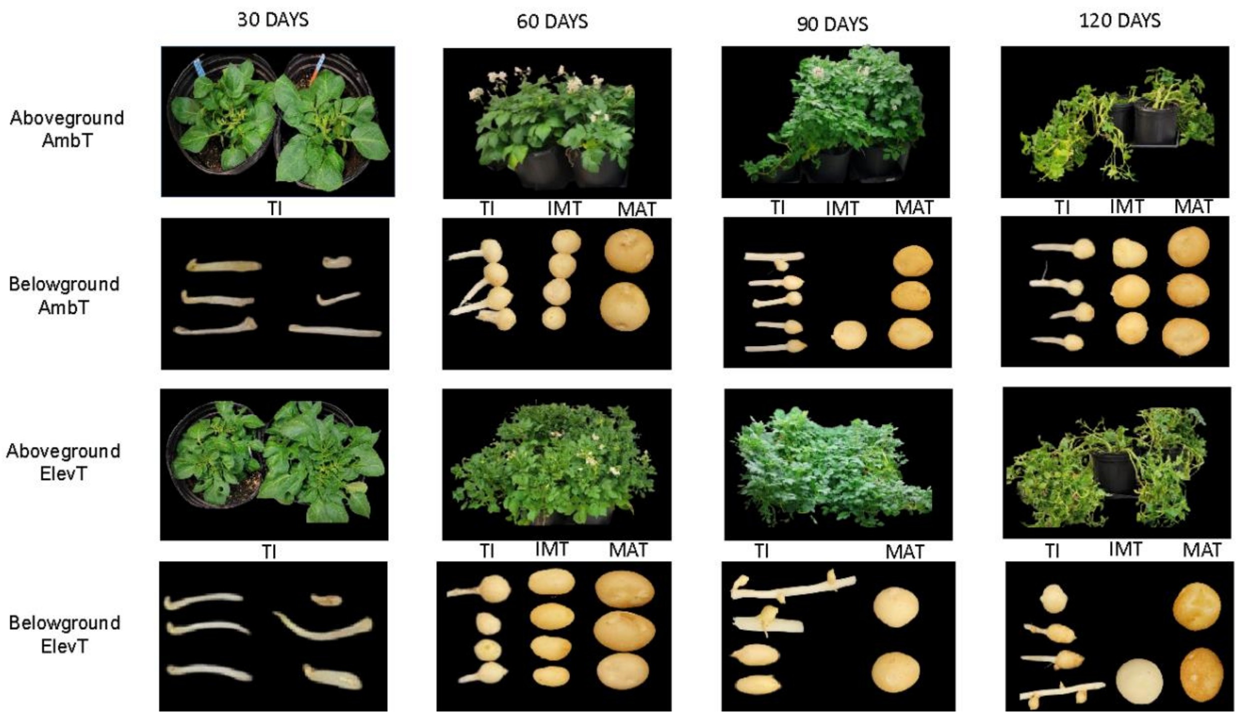

Supplementary Figure S3. Example of tuber size classes for this experiment. Photographs of tuber initials (TI), immature tubers (IMT), and mature tubers (MAT) collected from ambient (AmbT) and elevated temperature (ElevT) conditions throughout the growth chamber experiment. Tuber Initials (TI) (< 0.6g), Immature Tubers (IMT) (0.6-5g), and Mature Tubers (MAT) (> 5g). Images were digitally extracted for comparison.

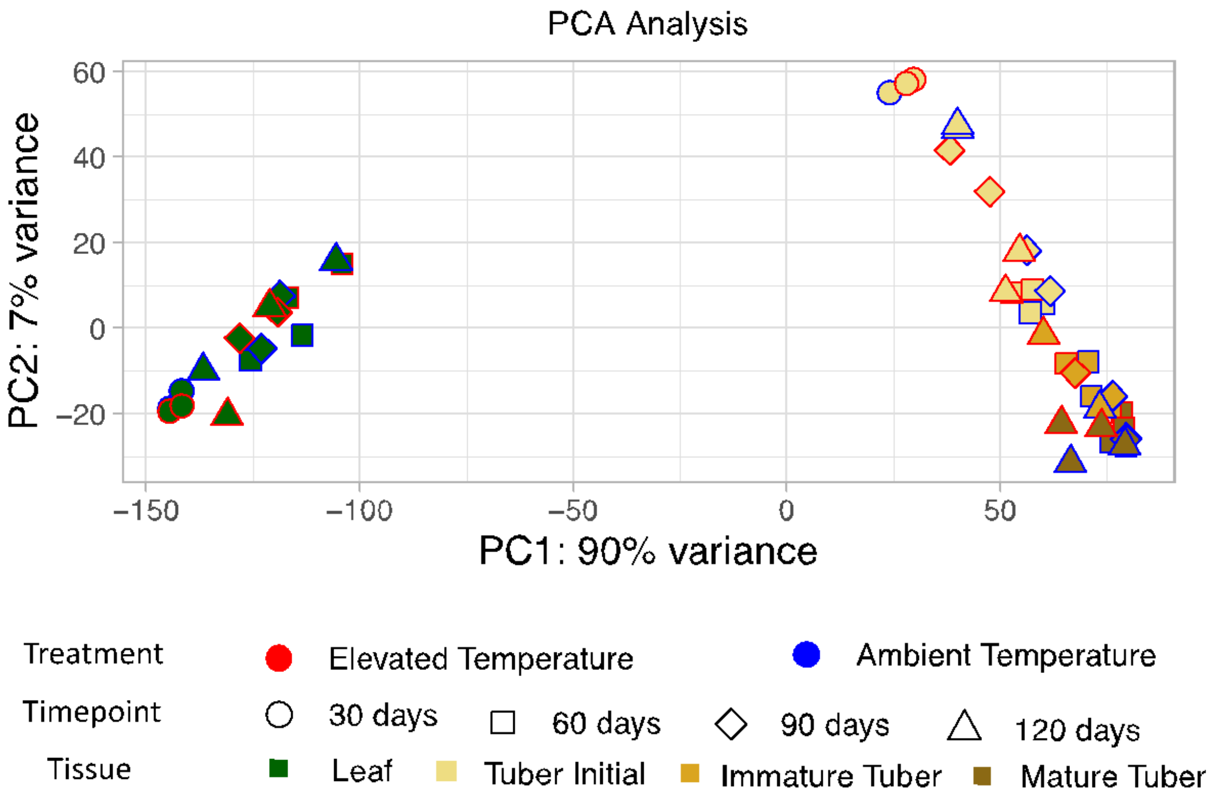

Supplementary Figure S4. Principal component analysis (PCA) of all RNA-seq libraries. Library samples are identified by treatment, timepoint and tissue. Graphs were produced using *ggplot* in R.

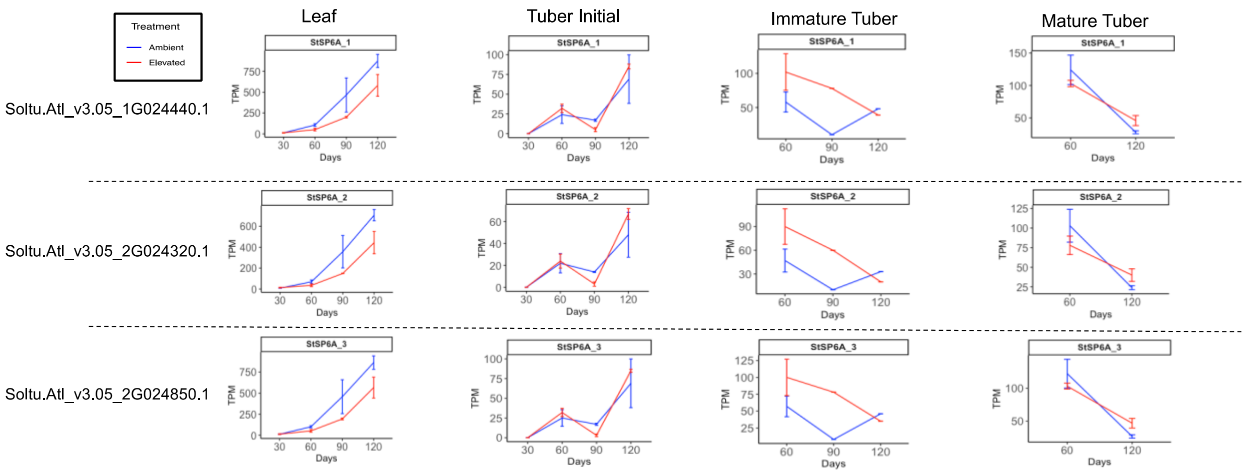

Supplementary Figure S5. Transcripts per million (TPM) values of the known tuberization promoting gene *StSP6A* syntelogs in each tissue type over 120 days. TPM values were determined from *Salmon* (Patro et al. 2017) and averaged per biological replicate (n = 1 or 2). Blue lines represent plants grown in ambient temperatures (AmbT) while red lines represent plants grown in projected elevated temperatures (ElevT). No ElevT mature tubers were collected at 90d, so no data is shown for that point. Error bars represent the standard error. Significance was determined by the Wald test using *DESeq2* (Love et al., 2014). *P*-values were adjusted for false discovery rate using a Benjamini-Hochberg correction method (* = $p < 0.10$; ** = $p < 0.05$).

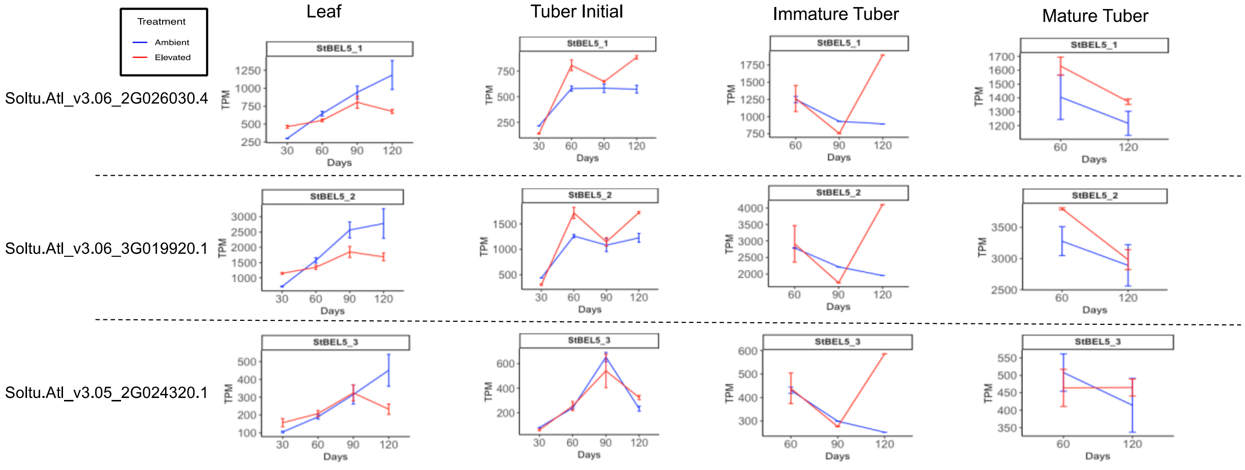

Supplementary Figure S6. Transcripts per million (TPM) values of the known tuberization promoting gene *StBEL5* syntelogs in each tissue type over 120 days. TPM values were determined from *Salmon* (Patro et al. 2017) and averaged per biological replicate (n = 1 or 2). Blue lines represent plants grown in ambient temperatures (AmbT) while red lines represent plants grown in projected elevated temperatures (ElevT). No ElevT mature tubers were collected at 90 d, so no data is shown for that point. Error bars represent the standard error. Significance was determined by the Wald test using *DESeq2* (Love et al., 2014). *P*-values were adjusted for false discovery rate using a Benjamini-Hochberg correction method (* = $p < 0.10$; ** = $p < 0.05$).

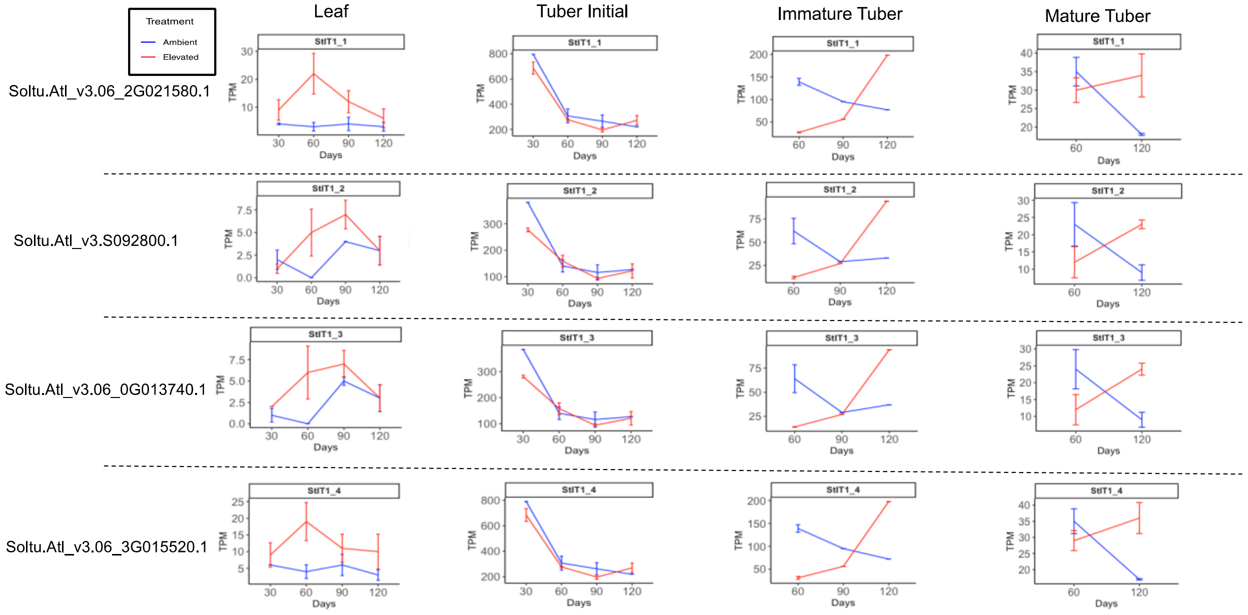

Supplementary Figure S7. Transcripts per million (TPM) values of the known tuberization promoting gene *StIT1* syntelogs in each tissue type over 120 days. TPM values were determined from *Salmon* (Patro et al. 2017) and averaged per biological replicate (n = 1 or 2). Blue lines represent plants grown in ambient temperatures (AmbT) while red lines represent plants grown in projected elevated temperatures (ElevT). No ElevT mature tubers were collected at 90 d, so no data is shown for that point. Error bars represent the standard error. Significance was determined by the Wald test using *DESeq2* (Love et al., 2014). *P*-values were adjusted for false discovery rate using a Benjamini-Hochberg correction method (* = $p < 0.10$; ** = $p < 0.05$).

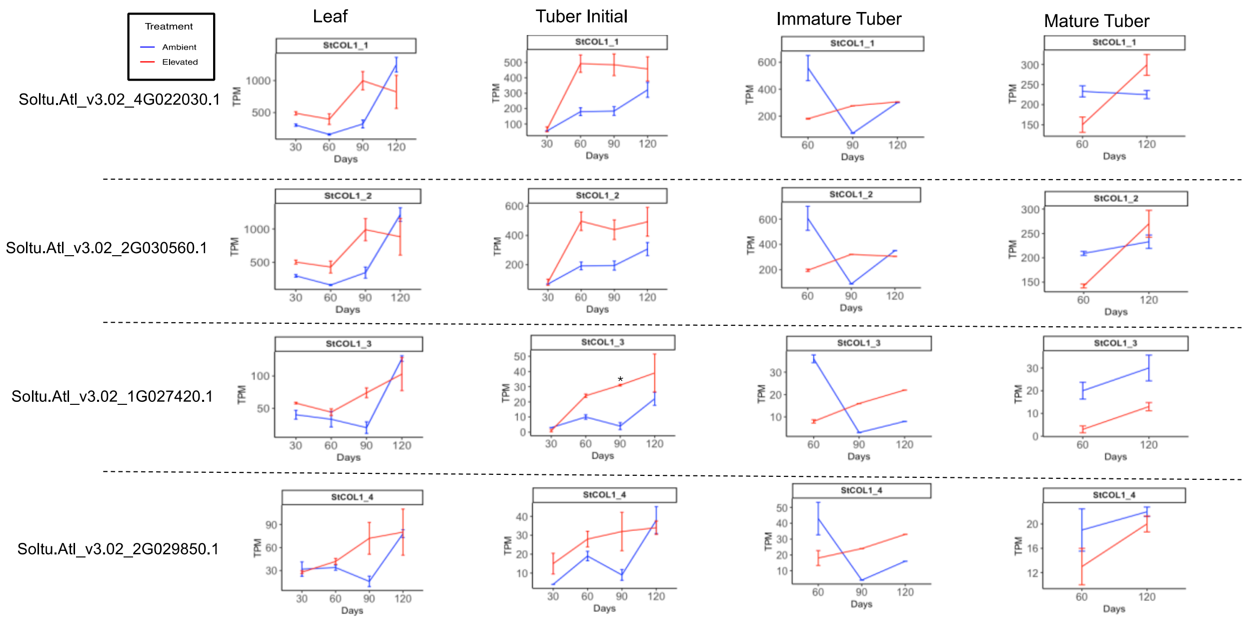

Supplementary Figure S8. Transcripts per million (TPM) values of the known tuberization inhibitor gene *StCOL1* syntelogs in each tissue type over 120 days. TPM values were determined from *Salmon* (Patro et al. 2017) and averaged per biological replicate (n = 1 or 2). Blue lines represent plants grown in ambient temperatures (AmbT) while red lines represent plants grown in projected elevated temperatures (ElevT). No ElevT mature tubers were collected at 90 d, so no data is shown for that point. Error bars represent the standard error. Significance was determined by the Wald test using *DESeq2* (Love et al., 2014). P-values were adjusted for false discovery rate using a Benjamini-Hochberg correction method (* = $p < 0.10$; ** = $p < 0.05$).

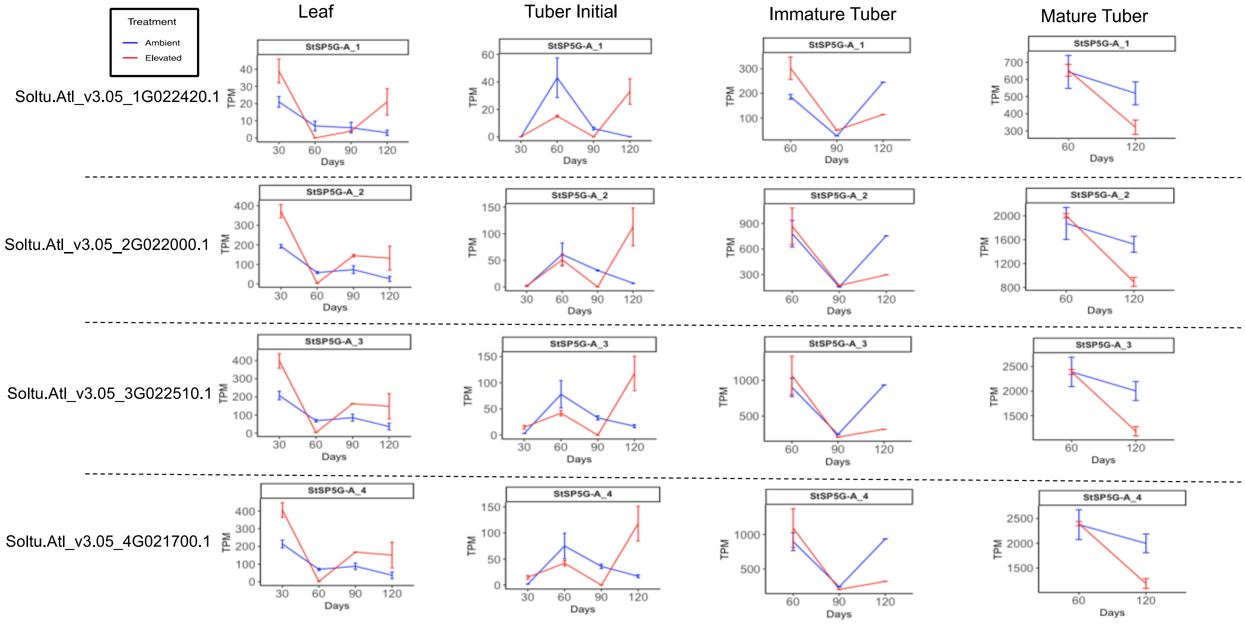

Supplementary Figure S9. Transcripts per million (TPM) values of the known tuberization inhibitor genes *StSP5G-A* syntelogs in each tissue type over 120 days. TPM values were determined from *Salmon* (Patro et al. 2017) and averaged per biological replicate (n = 1 or 2). Blue lines represent plants grown in ambient temperatures (AmbT) while red lines represent plants grown in projected elevated temperatures (ElevT). No ElevT mature tubers were collected at 90 d, so no data is shown for that point. Error bars represent the standard error. Significance was determined by the Wald test using *DESeq2* (Love et al., 2014). *P*-values were adjusted for false discovery rate using a Benjamini-Hochberg correction method (* = $p < 0.10$; ** = $p < 0.05$).

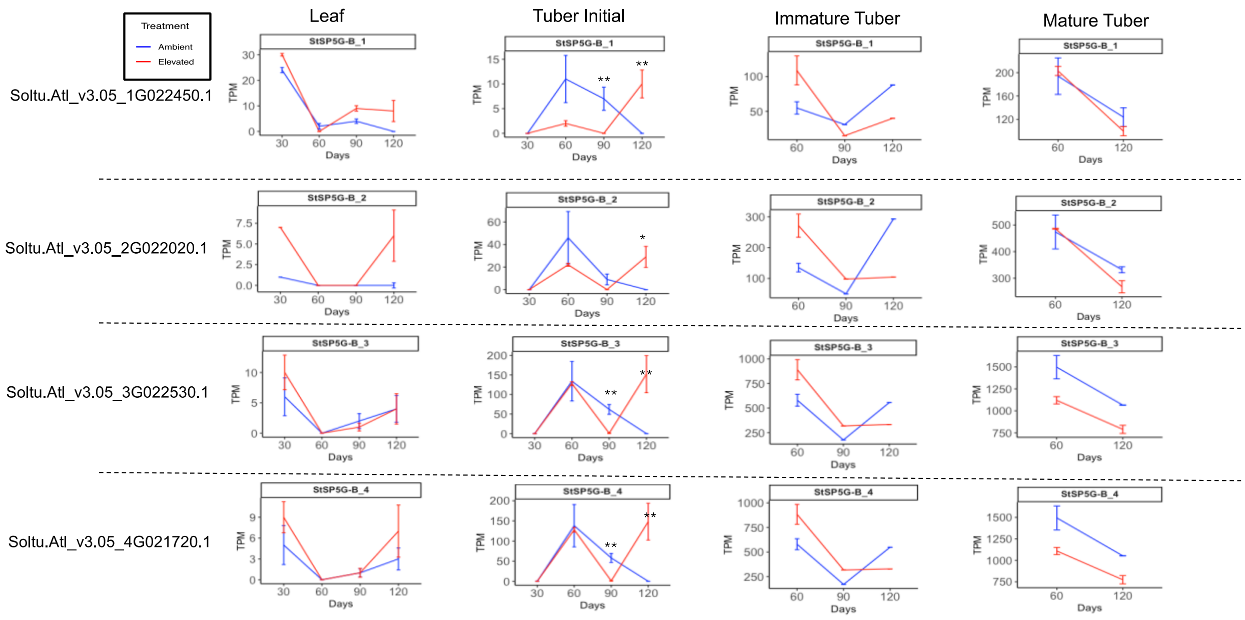

Supplementary Figure S10. Transcripts per million (TPM) values of the known tuberization inhibitor gene *StSP5G-B* syntelogs in each tissue type over 120 days. TPM values were determined from *Salmon* (Patro et al. 2017) and averaged per biological replicate (*n* = 1 or 2). Blue lines represent plants grown in ambient temperatures (AmbT) while red lines represent plants grown in projected elevated temperatures (ElevT). No ElevT mature tubers were collected at 90 d, so no data is shown for that point. Error bars represent the standard error. Significance was determined by the Wald test using *DESeq2* (Love et al., 2014). *P*-values were adjusted for false discovery rate using a Benjamini-Hochberg correction method (* = $p < 0.10$; ** = $p < 0.05$).

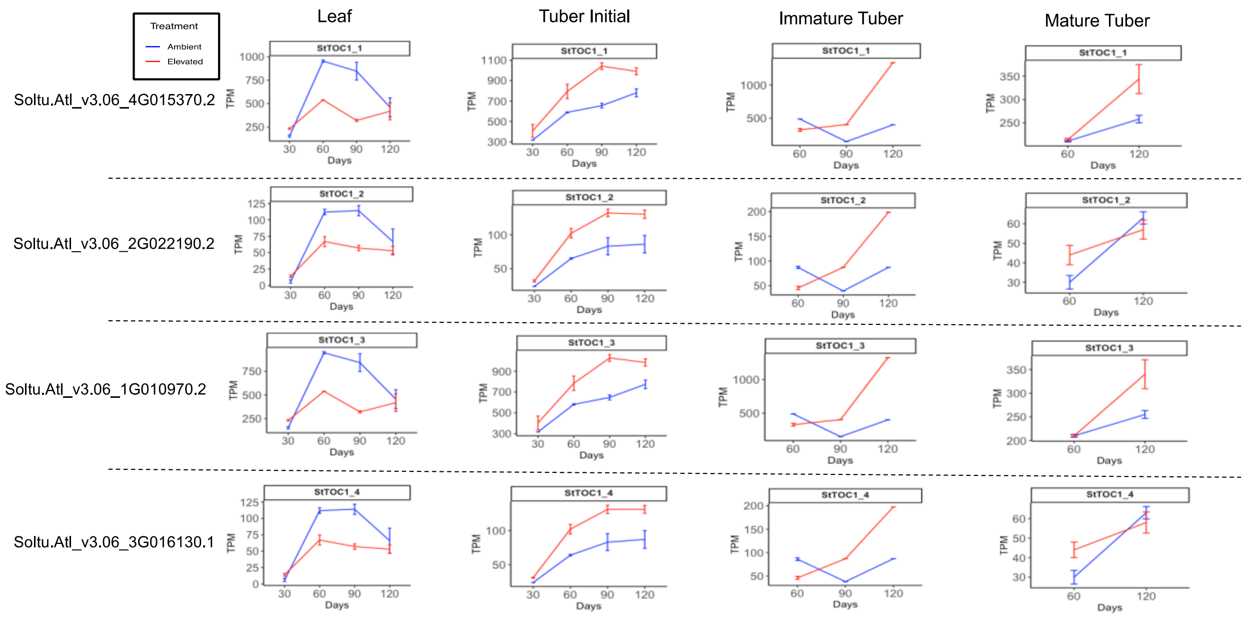

Supplementary Figure S11. Transcripts per million (TPM) values of the known tuberization inhibitor gene *StTOC1* syntelogs in each tissue type over 120 days. TPM values were determined from *Salmon* (Patro et al. 2017) and averaged per biological replicate (n = 1 or 2). Blue lines represent plants grown in ambient temperatures (AmbT) while red lines represent plants grown in projected elevated temperatures (ElevT). No ElevT mature tubers were collected at 90 d, so no data is shown for that point. Error bars represent the standard error. Significance was determined by the Wald test using *DESeq2* (Love et al., 2014). *P*-values were adjusted for false discovery rate using a Benjamini-Hochberg correction method (* = $p < 0.10$; ** = $p < 0.05$).
