# Supplementary material for: The physiological and molecular responses of potato tuberization to projected future elevated temperatures
Source: Plant Physiol. 2024 Dec 17;197(1):kiae664. doi: 10.1093/plphys/kiae664 (PMC11683837; doi:10.1093/plphys/kiae664)
Supplement: kiae664_Supplementary_Data [file pp.00921.2024-s10.pdf › pp.00921.2024-s10.pdf]

Plant Physiology®

Physiological and Molecular Responses of Projected Future Temperatures on Potato Tuberization

Abigail Guillemette, Guillian Hernández-Casanova, John Hamilton, Eva Pokorna, Petre Dobrev, Vaclav Motyka, Aaron Rashotte, and Courtney Leisner

Review Timeline:

Submission Date:	11-Jun-2024
Editorial Decision:	01-Oct-2024
Revision Received:	04-Nov-2024
Accepted:	20-Nov-2024

01-Oct-2024

Dr. Courtney P Leisner
Virginia Polytechnic Institute and State University
Blaskcburg, Virginia

RE: Physiological and Molecular Responses of Projected Future Temperatures on Potato Tuberization

Dear Dr. Courtney Leisner:

We are pleased to accept your manuscript for publication in the Plant Physiology. This acceptance is contingent on revision based on the comments of our reviewer. I strongly apologize for the long delay in the revision process, which was due to the lack of reviewers accepting the task for long. As you will see, there are comments from just one reviewer, but considering the expertise and clarity of his/her review I take the decision without waiting for a second reviewer whose review was due in August.

Please highlight all changes and include a detailed annotation to changes to the text, with line numbers, and noting your responses to the comments.

To submit your revised manuscript, click:

Link Not Available

If you cannot return the revised manuscript within 8 weeks of receipt, please let us know. Otherwise, we will assume that you have elected not to revise the manuscript and are withdrawing it.

Thank you for allowing us to review your work. We look forward to hearing from you soon.

Sincerely,

Editor, Plant Physiology

--- Reviewer comments:
Reviewer #2 (Comments for the Author):

This manuscript examines the effects of a realistically imposed heat stress over the growing period of potatoes to determine effects of elevated temperature on tuber development. The paper combines methods in physiology, plant hormones, and gene expression to better describe where heat stress, in terms of development, affects tuber growth. The manuscript is clearly written and easy to follow.

A list of specific, line by line comments:

L43 - "develop" instead of "development"
L61 - "have a shift" instead of "face." Or "demonstrate"?
L67 - "affect"
L96 - these two genes are introduced here without full names or any other context, a bit abruptly.

The introduction discusses known regulators of tuber formation and often addresses how those regulators respond to heat stress. I know there is much to be learned here still, but the current set up undersells the advancement of your study. Perhaps, in sections where you describe gene X has increased/decreased expression under heat and affects gene Y, you point out what knowledge gaps still remain?

L125-128 - can you specify the exact temperatures used? Oh - I see it's further down. Up to you but feels like it might fit better in this first paragraph.

L162 "were set"

L189 - why was this done only at 90 and 120 d?
L190 - duplicate "and collected"
L192 - "ground" instead of "grounded"

L282 - this means you averaged TPM values for expression in each treatment separately for leaf and tuber, yes? It's confusing as written. Perhaps "at each timepoint for the two separate tissues."?

L283 - What are "TI" and "IMT"?

What proportion of genes of interest were duplicated in the ATL genome relative to DM? What is the downside to analyzing all genes separately, besides the difficulty of plotting, etc? It's hard to know how much within syntelog variation was removed by taking an average.

L382 - do you mean multiple test correcting?

L393 - this feels like a big result that is just cast to be seen only in a figure - perhaps more text here is warranted? Especially since many of the canonical tuberization genes did not show differential expression between treatments.

L400 - how did you pick which syntelog to show? This is fine, so long as the chosen syntelogs were chosen systematically (eg ones with highest expression, or largest difference in treatments)

L417 - "decreased" - some tense mismatch in this section

Figure 5 - It was not clear from the methods that the phytohormone samples were pooled across 90 and 120 d old plants - why was that done? Isn't it possible it's obscuring patterns?

Response to Review
We are writing in response to the review of our manuscript entitled "Physiological and Molecular Responses of Projected Future Temperatures on Potato Tuberization", manuscript #PP2024-RA-00921. We have read over the editor and reviewer comments and hereby submitting a revised manuscript. We believe the revised manuscript addresses all reviewer comments and now fully meets the criteria for publication in Plant Physiology. We have addressed each comment from the reviewer below.

Reviewer #2 (Comments for the Author):
This manuscript examines the effects of a realistically imposed heat stress over the growing period of potatoes to determine effects of elevated temperature on tuber development. The paper combines methods in physiology, plant hormones, and gene expression to better describe where heat stress, in terms of development, affects tuber growth. The manuscript is clearly written and easy to follow.

A list of specific, line by line comments:

L43 - "develop" instead of "development"
This edit has been made.

L61 - "have a shift" instead of "face." Or "demonstrate"?
This edit has been made to "demonstrate".

L67 - "affect"
This edit has been made.

L96 - these two genes are introduced here without full names or any other context, a bit abruptly.
We have edited the text to read "The CONSTANS LIKE (StCOL1) and Self Pruning (StSP5G) inhibitory pathway is known to be an important suppressor of tuberization".

The introduction discusses known regulators of tuber formation and often addresses how those regulators respond to heat stress. I know there is much to be learned here still, but the current set up undersells the advancement of your study. Perhaps, in sections where you describe gene X has increased/decreased expression under heat and affects gene Y, you point out what knowledge gaps still remain?
We appreciate this feedback from the reviewer. We have added language in the discussion to further describe how our findings fit into our current understanding of heat stress impacts on tuberization in potato and what knowledge gaps are remaining.

L125-128 - can you specify the exact temperatures used? Oh - I see it's further down. Up to you but feels like it might fit better in this first paragraph.
We have moved up the description of the exact temperatures used per the reviewer's suggestion.

L162 "were set"
This edit has been made.

L189 - why was this done only at 90 and 120 d?
Samples were only analyzed at 90 and 120 days due to cost limitations. Due to the number of tissues, replicates and treatments, we limited analysis only to the time points we thought might be most valuable based on the physiology data.

L190 - duplicate "and collected"
This edit has been made.

L192 - "ground" instead of "grounded"
This edit has been made.

L282 - this means you averaged TPM values for expression in each treatment separately for leaf and tuber, yes? It's confusing as written. Perhaps "at each timepoint for the two separate tissues."?
Thank you for pointing this out. We have updated the statement for clarity. It now reads "From our tissue sampling, we then averaged TPM values for each syntelog for each tissue type at every timepoint (n = 2)."

L283 - What are "TI" and "IMT"?
The definitions for these abbreviations are given in the manuscript on lines 206-207.

What proportion of genes of interest were duplicated in the ATL genome relative to DM? What is the downside to analyzing all genes separately, besides the difficulty of plotting, etc? It's hard to know how much within syntelog variation was removed by taking an average.
We thank the review for the questions. There is syntelog-specific expression demonstrated in Atlantic. Previous work in Hoopes et al., (2022) has demonstrated there is an average of 3.2 copies per gene in the ATL genome verses the diploid monoploid (DM) genome. Among Atlantic tetra-allelic groups, 56.2% and 82.7% have four distinct CDS and cDNA sequences, respectively. Another 36.1% and 14.8% have three distinct CDS and cDNA sequences, respectively. It has also been demonstrated that there is a preferential syntelog expression in Atlantic and a total of 42.8% and 41.2% of the allelic groups displaying preferential expression patterns in Atlantic leaf and tuber tissue, respectively, contained deleterious/dysfunctional alleles. In our paper we chose to describe syntelog-specific expression and presented this in both main text and supplemental figures. The only average that was taken was across biological replicates for each syntelog, which is demonstrated by error bars in the line plots, accounting for within syntelog variation across biological replicates.

Reference: Hoopes et al., (2022) ; https://www.sciencedirect.com/science/article/pii/S167420522200003X

L382 - do you mean multiple test correcting?
We have changed the text from "p-adjusted" to "Genes with adjusted p value of < 0.05 using a Bonferroni correction method" to provide clarity.

L393 - this feels like a big result that is just cast to be seen only in a figure - perhaps more text here is warranted? Especially since many of the canonical tuberization genes did not show differential expression between treatments.
We have added more language to the results to describe the differentially expressed genes that we saw in the 60d and 90d timepoints that related to the physiological data analysis that was completed.

L400 - how did you pick which syntelog to show? This is fine, so long as the chosen syntelogs were chosen systematically (eg ones with highest expression, or largest difference in treatments).
As stated in the text, syntelogs graphed in Figure 7 in the manuscript represent syntelog(s) found to be differentially expressed or those with highest transcript abundance while all syntelogs from the genes described are in Supplementary Figures 5A-G.

L417 - "decreased" - some tense mismatch in this section.
This edit has been made.

Figure 5 - It was not clear from the methods that the phytohormone samples were pooled across 90 and 120 d old plants - why was that done? Isn't it possible it's obscuring patterns?
We have updated the legend for Figure 5 to indicate that samples were pooled across 90 and 120 old plants that had similar trends. Supplemental data for each time point (90d and 120d) is provided in Supplemental Table 5A-D. Analysis phytohormones at 90d and 120d individually demonstrated similar expression patterns. Further statistical analysis was done using pooled samples increase the number of biological replicates we had per tissue type at each time point in both ambient and elevated temperature treatments.

20-Nov-2024
Dr. Courtney P Leisner
Virginia Polytechnic Institute and State University
Blaskcburg, Virginia

MSID: PP2024-RA-00921R1
MS TITLE: Physiological and Molecular Responses of Projected Future Temperatures on Potato Tuberization

Dear Dr. Courtney Leisner:

I am pleased to inform you that your revised manuscript "Physiological and Molecular Responses of Projected Future Temperatures on Potato Tuberization" has been accepted for publication in Plant Physiology. Your manuscript will be checked for consistency with journal formatting and image standards by one of our Science Editors, who will contact you shortly. They may have a number of queries and suggestions for you. Please keep in mind that they work to increase the online visibility of your article and its attraction for readers from the widest of backgrounds. You will have the opportunity to review any revisions, and I hope you will work constructively with them to get the most out of your research. You will also have the opportunity to review all changes when you receive the author proofs for your article.

Once validated for publication, your article will be sent to a Science Editor for a final review of your manuscript and source files. After your final files are uploaded, your article will appear in the next available issue and the online Preview version of your article will be posted after your signed Copyright and Checklist forms and final source files are received.

Please be sure to include the following in your manuscript file:
Harvard style citations
List of all author contributions and funding information at the beginning of manuscript after the titles and author list.
List all supplemental materials associated with the article, with titles, at the end of the ms, before the references and acknowledgments section.
Complete Figure Legends for all figures appearing in the article listed before the Literature Cited.

LICENSE INFORMATION Shortly after your paper is sent to production, you will receive an email from our publisher's author support team, **SciPris**, with information on article processing charges, as well as the types of Open Access license you would be able to purchase. The accepted version of your manuscript will not appear on our Advance Articles page until the license is selected and signed, so please look out for an email from SciPris. For more information see the Article Publication Fees section of our author guidelines: https://academic.oup.com/plcell/pages/General-Instructions

Finally, we encourage your submission of artwork for the journal cover. If there is an image or illustration related to your paper that you would like to have considered, please email it in .pdf or .tif format to clowe@aspb.org. An image title, brief, 2-3 sentence description of the image, and the name of the person credited for the image are also encouraged. If your image is selected to appear on the online cover of the journal, you will be contacted with the scheduled issue date and further information.

Thank you and congratulations on your Plant Physiology paper!

Sincerely,
Editor, Plant Physiology

=====
FIRST AUTHOR PROFILES
=====

We recognize the hard work that goes into being First Author of a Plant Physiology paper by publishing a "First Author Feature" for each First Author alongside your paper.

If the first author(s) would like to submit information for a feature on Facebook and Twitter, fill out the form by clicking on the link below:

Plant Physiology First Author Feature Form: https://forms.office.com/r/ZBCGZfhm8F

If you cannot access the form through the link above, please contact Rachel at rbelsky@aspb.org.

To upload an image of yourself to accompany your profile, please put your photo in the correct journal folder at the link below. You must label your image: Author First Name Last Name Article Title.

First Author Profile Image Dropbox: https://www.dropbox.com/scl/fo/xz029p7qjr2jml2rp2kmq/h?dl=0&rlkey=6fkxcaqmxx35ap152b2osy1fz

=====
IMPORTANT REMINDER: PEER REVIEW REPORTS
=====

If you opted to publish a peer review report along with your article during the original submission process, it will be prepared by the editorial staff and publicly posted with your manuscript, inside the zip file that contains any other supplemental material. As a reminder, the peer review report is a public record of all comments from editors and reviewers, as well as your prior responses, as you received them in the decision letters for each draft of your manuscript. If you agreed to publish this report and have changed your mind, or are not sure if you selected this option, please contact the editorial office as soon as possible before signing the license agreement from our publisher.

--- Reviewer comments:
